# Insights into the *Alcyoneusvirus* Adsorption Complex

**DOI:** 10.3390/ijms24119320

**Published:** 2023-05-26

**Authors:** Algirdas Noreika, Rasa Rutkiene, Irena Dumalakienė, Rita Vilienė, Audrius Laurynėnas, Simona Povilonienė, Martynas Skapas, Rolandas Meškys, Laura Kaliniene

**Affiliations:** 1 Department of Molecular Microbiology and Biotechnology, Institute of Biochemistry, Life Sciences Center, Vilnius University, Saulėtekio Av. 7, LT-10257 Vilnius, Lithuania; algirdas.noreika@gmc.vu.lt (A.N.);; 2 Department of Immunology, State Research Institute Center for Innovative Medicine, Santariškių St. 5, LT-08410 Vilnius, Lithuania; 3 Department of Bioanalysis, Institute of Biochemistry, Life Sciences Center, Vilnius University, Saulėtekio Av. 7, LT-10257 Vilnius, Lithuania; 4 Department of Characterisation of Materials Structure, Center for Physical Sciences and Technology, Saulėtekio Av. 3, LT-10257 Vilnius, Lithuania

**Keywords:** *Klebsiella pneumoniae* phage, vB_KleM_RaK2, *Alcyoneusvirus*, adsorption, tail fibers

## Abstract

The structures of the Caudovirales phage tails are key factors in determining the host specificity of these viruses. However, because of the enormous structural diversity, the molecular anatomy of the host recognition apparatus has been elucidated in only a number of phages. *Klebsiella* viruses vB_KleM_RaK2 (RaK2) and phiK64-1, which form a new genus *Alcyoneusvirus* according to the ICTV, have perhaps one of the most structurally sophisticated adsorption complexes of all tailed viruses described to date. Here, to gain insight into the early steps of the alcyoneusvirus infection process, the adsorption apparatus of bacteriophage RaK2 is studied in silico and in vitro. We experimentally demonstrate that ten proteins, gp098 and gp526–gp534, previously designated as putative structural/tail fiber proteins (TFPs), are present in the adsorption complex of RaK2. We show that two of these proteins, gp098 and gp531, are essential for attaching to *Klebsiella pneumoniae* KV-3 cells: gp531 is an active depolymerase that recognizes and degrades the capsule of this particular host, while gp098 is a secondary receptor-binding protein that requires the coordinated action of gp531. Finally, we demonstrate that RaK2 long tail fibers consist of nine TFPs, seven of which are depolymerases, and propose a model for their assembly.

## 1. Introduction

For most known viruses, one of the key steps in the infectious cycle is the adsorption of a virus particle onto the surface of a sensitive cell [1,2]. In the case of tailed bacterial viruses, i.e., phages that belong to the class Caudoviricetes, adsorption is mediated by receptor-binding proteins (RBPs), such as tail spike, tail fiber, or tail tip proteins, usually located at the distal part of the tail [3,4,5]. Phage adsorption is a two-step process [5,6]. When a phage particle encounters a viable permissive cell, viral RBPs first reversibly bind to the primary cell surface receptors. Then, if physicochemical conditions are favorable, either the same or secondary RBPs irreversibly bind to secondary host cell receptors (in some cases, a different moiety on the primary receptor), leading to permanent structural changes in the virion and DNA ejection [4,5,6,7].

Tailed phages show remarkable structural diversity and use numerous different mechanisms for adsorption [5,6]. Based on the tail morphology, these phages can be grouped into three distinct morphotypes: (i) siphoviruses with long noncontractile tails, (ii) podoviruses with short noncontractile tails, and (iii) myoviruses that possess thick, contractile tails [8,9]. The latter group, i.e., myoviruses, have the most sophisticated multi-molecular adsorption apparatus [10,11]. Perhaps the best-studied myovirus is *Escherichia coli* phage T4, which uses two types of tail fibers to adsorb to its host cell. First, T4 long tail fibers, formed by gp34–37, bind reversibly to lipopolysaccharides (LPS) or the outer membrane protein OmpC [10,11,12]. This causes a significant change in the conformation of the baseplate so that the short tail fibers (gp12), which are folded in the pre-attachment baseplate, stretch out and fully extend to reach their receptor, the outer core region of LPS. The short tail fibers irreversibly bind to the cell surface and orient the phage particle for DNA ejection [10,11,12]. Not all myophages have a baseplate as complex as that of T4, nor do they all use the same host adsorption mechanism [10,11]. For example, myoviruses belonging to the *Ackermannviridae* family, such as O-antigen-specific *E. coli* phage CBA120 or *Salmonella* phage Det7, encode short and stubby tail fibers formed by the enzymatic RBPs called tail spike proteins (TSPs) [13,14,15]. Remarkably, bacteriophage Det7 cannot adsorb to rough bacterial strains since its genome ejection is triggered by the binding of TSPs to bacterial LPS and its subsequent enzymatic degradation [16].

Many tailed phages, such as *Klebsiella*, *Acinetobacter*, or *Salmonella*-targeting viruses, have adapted to use cell surface polysaccharides (i.e., exopolysaccharides, capsular saccharides, or LPS) as primary receptors [17,18,19]. An important structural component of the adsorption apparatus of such phages is enzymatic RBPs, commonly known as depolymerases. Phage depolymerases, biochemically subdivided into hydrolases and lyases, specifically recognize and cleave the cell surface polysaccharides to provide access to the cell wall or to the secondary phage receptors [4,18,19,20,21]. Although phage depolymerases are highly diverse in their amino acid sequence, their modular structure is quite conserved. Depolymerases are generally homotrimers and contain three domains: an N-terminal particle-binding domain, a central highly specific polysaccharide-degrading domain, and a C-terminal domain, which in some RBPs promotes oligomerization and/or host binding [17,18,19,20,21,22,23]. Most known phages encode only one or two depolymerases and have simple unbranching receptor-binding structures attached to the distal part of their tails [14,18,19,24]. However, some phages encode multiple enzymatic RBPs and possess branching tail fibers where each branch is likely formed by a single protein [19,25,26]. Interestingly, based on the results of bioinformatic and structural analyses, it has been shown recently that phages assemble their branching tail fibers using conserved N-terminal domains, which often show structural similarity to those of the baseplate proteins of bacteriophage T4 [14,19,26,27,28].

The *E. coli* phage CBA120 has perhaps the best-studied branching tail fibers, consisting of four TSPs (TSP1–TSP4) that recognize and degrade different O-antigen serotypes (Figure S1). Each TSP can be broadly divided into two main regions: the N-terminal head region comprising two homologous domains, D1 and D2 (only D1 in the case of TSP2) and the C-terminal body region, which contains an enzymatic domain (ED) and a domain D4 of unknown function [27,29,30,31,32]. In addition, the N-termini of TSP2 and TSP4 contain two and three T4 gp10-like domains (XD), respectively, and both proteins have a small D3’ domain inserted between the head and body. It has been shown recently that the enzymatic β-helical domains of CBA120 TSPs are involved in O-antigen binding and subsequent degradation, while conserved N-terminal domains mediate the assembly of the branching receptor-binding complex, which is attached to the baseplate via the small N-terminal “anchor” domain (AD) of TSP4 [27,29,30,31,32]. Overall, the structure of the CBA120 TSPs illustrates how even phylogenetically distant phages use evolutionarily proven pathways and modules to form molecular complexes of similar but not identical functions. It is also evident that the modular architecture of the RBPs enables domain swapping through horizontal gene transfer, allowing phage host range expansion [19,25,27,33].

In our previous studies, we described a unique jumbo myovirus RaK2 that possesses probably the most elaborate branched adsorption complex [34,35]. Bioinformatics and MS/MS analyses indicated that at least ten putative structural proteins (gp098 and gp526–gp534) are responsible for the formation of the six long RaK2 tail fibers. At the time, RaK2 had been a singleton phage, however, a close relative of similar morphology, a giant *Klebsiella* myovirus phiK64-1, was isolated and published a few years later [36,37]. Bacteriophage phiK64-1 was predicted to encode 11 TFPs that exhibited 26–100% sequence identity to the aforementioned TFPs of RaK2 (Table S1), suggesting that these two viruses may have at least partially overlapping host ranges. A total of nine TFPs of phiK64-1 (Table S1) were functionally characterized and were shown to possess depolymerase activity against ten capsular serotypes of *Klebsiella*, namely, K1, K11, K21, K25, K30, K35, K64, K69, KN4, and KN5 [36,37]. Nevertheless, the molecular anatomy of the adsorption apparatus has not yet been investigated for either phiK64-1 or RaK2, the two extraordinary viruses that now form the genus *Alcyoneusvirus* [38].

Here, to gain insight into the early steps of the *Alcyoneusvirus* infection process at the molecular level, the ten proteins predicted to form the elaborate tail fibers of RaK2 were analyzed by combining in silico and in vitro techniques. We experimentally confirmed the presence of all ten RaK2 TFPs in the RaK2 adsorption apparatus and showed that two of these proteins, gp098 and gp531, are essential for the recognition and attachment of K. pneumoniae KV-3 cells. We demonstrated that the long tail fibers of RaK2 are composed of nine TFPs, seven of which contain a polysaccharide-degrading catalytic domain, yet during *K. pneumoniae KV-3* infection, only gp531 acts as a primary RBP, which recognizes and degrades the capsule of the host. The product of RaK2 g*098*, which is not a component of the long tail fibers but a baseplate protein, likely functions as a secondary RBP and requires gp531 to act in concert during adsorption onto the KV-3 cell surface.

## 2. Results

Previously we suggested that the elaborate and highly complex long tail fibers of the phage RaK2 may be formed from at least ten proteins (gp098, gp526–gp534), all of which, according to MS/MS, are present in mature virus particles [34,35]. The genes for nine of these proteins (gp526–gp534) are organized into a ~19 kb cluster located ~20 kb upstream of the other RaK2 structural genes. Meanwhile, g*098* is adjacent to the baseplate/tail/capsid module, ~230 kb upstream of the tail fiber gene cluster. However, in the absence of close relationships with proteins of known function and structure, where even HHpred, which can detect distant homologies [39], fails to find significant matches for the entire length of the RaK2 TFPs, it is difficult to predict which of these proteins are the actual components of the RaK2 tail fibers or to infer their biological function. Therefore, in this study, we combined in silico analysis with biological experiments to shed light on the architecture of the phage RaK2 adsorption complex and to elucidate the role of individual proteins in host-attachment.

### 2.1. In Silico Analysis of RaK2 TFPs

To gain insight into the structure of the individual RaK2 TFPs, we used AlphaFold2, which was shown to build high-confidence 3D models of proteins [40,41,42]. Since most known tail fiber and tail spike proteins function as trimers [10], all TFPs were modeled as monomers and homotrimers. The predicted structures of the individual domains were then submitted to the Dali [43,44] server to search for possible structural relationships with distantly related characterized proteins.

**Figure 1:**
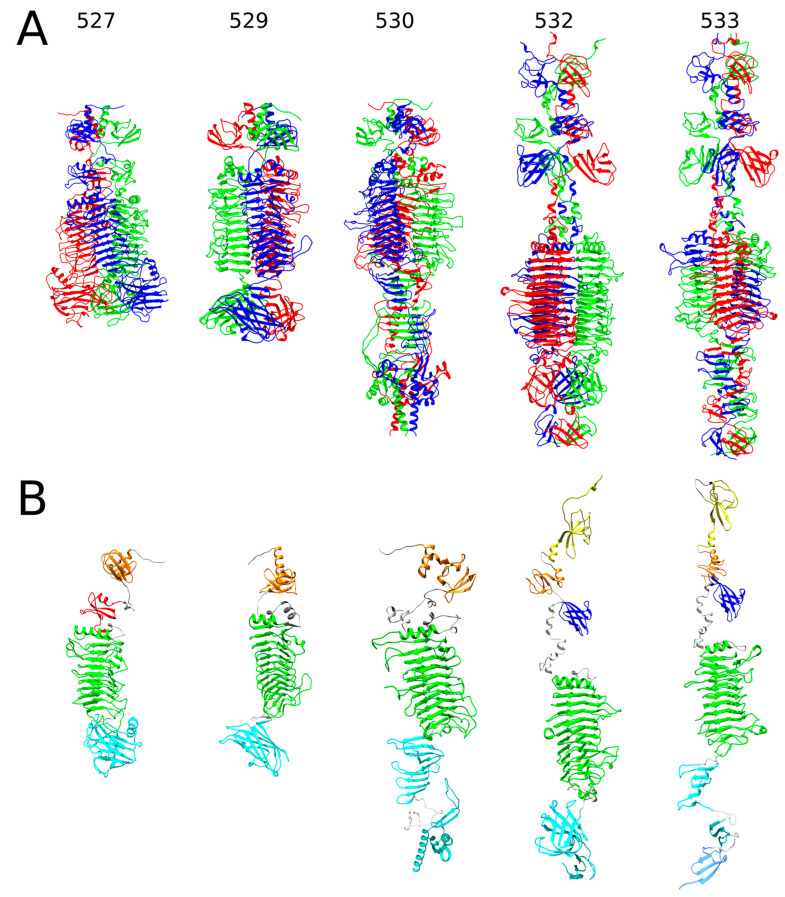
AlphaFold2-generated 3D models of RaK2 gp527, gp529, gp530, gp532, and gp533. (**A**) Ribbon representation of predicted trimeric assemblies; the individual monomers are colored in red, green, and blue. (**B**) Ribbon representation of monomers obtained from the predicted 3D structures of trimers. The N-terminal domains are colored based on their similarity to corresponding domains of CBA120 TSPs: orange—D1, yellow—D2, red—D3′, and blue—XD2. The catalytic depolymerase domains are colored green, and the C-terminal domains are cyan and light blue; domain linkers are colored gray.

For five of the ten RaK2 TFPs studied (gp527, gp529, gp530, gp532, and gp533), AlphaFold2 prediction yielded an elongated trimeric structure, which is broadly subdivided into an N-terminal head-like region and a body-like region, i.e., a shape typical to phage TSPs with depolymerase activity (Figure 1A and Figure S2). However, the structural regions of these five proteins differ in length and the number of domains they contain, which is particularly evident in the cartoon representation of monomers (Figure 1B).

The AlphaFold2-generated 3D models suggested that the N-termini of RaK2 gp529 and gp530 each fold into a single domain consisting of an α-helix and a six-stranded β-sandwich, followed by a linker of 34 and 63 residues, respectively. In the case of gp529, the N-terminal domain was identified by Dali (Table S2) as being similar to D1 of CBA120 TSP1 (PDB ID: 4oj5) [30], whereas a corresponding domain of gp530 is structurally closer to D1 of TSP4 (5w6h [27]). Of note, D1 domains of the four CBA120 TSPs have the same fold and superpose fairly well [27,31]. For both gp529 and gp530, AlphaFold2 predicted a central right-handed parallel β helix domain (CD) capped by a three-turn α-helix (Figure 1), a fold common to diverse polysaccharide-degrading proteins [45,46]. The CD of gp529 has 12 rungs and shows similarity to a poly(beta-D-mannuronate) C5 epimerase 6 from *Azotobacter vinelandii* (5lw3) and to the catalytic domain of CBA120 TSP2 (5w6s [31]). Meanwhile the predicted structure of the central β-helical domain of RaK2 gp530 (residues 151–504), which has 11 rungs, is similar to the endorhamnosidase domain of *Shigella flexneri* phage Sf6 TSP (2vbm [47]). Unexpectedly, according to Dali, the top match to the predicted structure of gp529 C-terminal domain (residues 448–584) is that of *E. coli* phage T4 gp9 (1qex [48]). In contrast, the similarity scores with the next match, the C-terminus of *Acinetobacter baumanii* phage AM24 tail spike protein 2 (5w5p), were much lower. T4 gp9 is a baseplate protein, whose C-terminal eight-stranded antiparallel β-barrel domain (residues 167–288) serves as an attachment site for the long tail fibers (via the N-terminus of gp34) and is also responsible for proper folding and trimerization of the polypeptide chains [48,49,50]. Therefore, it is likely that the C-terminal domain of gp529 facilitates trimerization of the protein as well. Based on the AlphaFold2 prediction, the C-terminal region of gp530 is composed of two domains, a CTD1 (comprising two triangular β-prisms, aa 512–627) and a CTD2 (intra-molecular chaperone, aa 653–779). The second prism and the CTD2 of gp530, residues 579–779, show similarity to the carboxy-terminal region of the bacteriophage T5 L-shaped tail fiber (4uw8 [51]). In T5, an intra-molecular chaperone is auto-proteolyzed after facilitating the correct folding of the TFP, and the remaining receptor-binding domain recognizes oligo-mannose [51]. According to our results (see Section 2.2), the ~14 kDa peptide is auto-proteolytically removed from the C-terminus of gp530 during protein overexpression, suggesting that this RaK2 TFP also has an active intra-molecular chaperone.

AlphaFold2 predicted that the N-terminus of gp527 (181 aa) of RaK2 comprises two domains: NTD I, which has the same fold as D1 of TSP1 (4oj5 [30]), and NTD II, which adopts a fold of four-stranded open face β-sandwich wrapped around a two-turn α-helix and which, according to Dali, is structurally the most similar to D3’ of TSP2 (5w6p [31]). The body region of gp527 (residues 176–716), encompassing the catalytic right-handed parallel β helix domain and the C-terminal β-sandwich domain, is structurally the most similar to that of the TSP4 (5w6h [27]) from *E. coli* phage CBA120.

Figure 1 shows that the head regions of the RaK2 gp532 and gp533 3D models include three domains (NTD1-3) separated from the body by an α-helical neck. For both RaK2 TFPs, NTD1 and NTD2 domains show similarity to D2 and D1 from the CBA120 TSPs, respectively, while NTD3 is structurally similar to XD2 from the CBA120 TSP4 (Table S2). The predicted body regions of gp532 (residues 281–806) and gp533 (residues 284–767) contain three (CD and CTD1-2) and four domains (CD and CTD1-3), respectively. As seen in Table S2, Dali returned the central β-helical domain of the *Bacillus* virus phi29 preneck appendage protein gp12 (3suc [52]) as a hit for the CD of gp532 and the enzymatic domain of CBA120 TSP2 (5w6s [31]) as a match to the RaK2 gp533 CD. However, in both cases, the top match was the poly(beta-D-mannuronate) C5 epimerase 6 (5LW3) from *Azotobacter vinelandii*. A Dali search of the CTDs of gp532 and gp533 revealed no significant structural similarity (high Z-value, low RMSD) with any PDB entries of phage origin, with the possible exception of CDT1 of gp533, which has a similar fold to the C-terminus of the TSP of Salmonella phage P22 (2XC1 [53]). Notably, according to HHpred, the entire C-terminal region of gp533 shows similarity to that of CBA120 TSP2 (Table S2).

**Figure 2:**
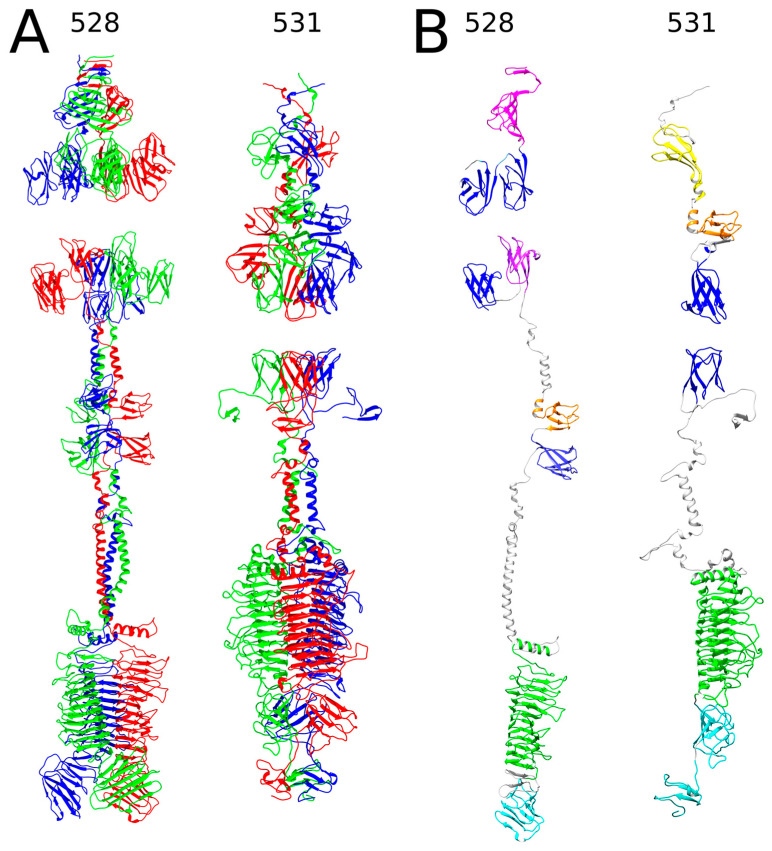
The predicted structures of RaK2 gp528 and gp531. (**A**) Ribbon representation of predicted trimeric assemblies; the individual monomers are colored in red, green, and blue. (**B**) Ribbon representation of monomers obtained from the predicted 3D structures of trimers. The color code is as follows: pink—anchor domain (AD), orange—D1, yellow—D2, blue—XD2, purple—XD3, green—the catalytic domain, cyan—CTDs, gray—linker. Note that in the monomeric structures, overlapping regions with lower pLDDT values were removed for clarity.

Two other RaK2 TFPs, gp528 and gp531, were also predicted to contain a polysaccharide-degrading parallel β-helix domain. However, as shown in Figure 2, likely owing to the lack of solved structures with similar amino acid sequence and domain composition, AlphaFold2 could not assemble the N-terminal domains of gp528 and gp531 in the correct orientation and provide accurate and meaningful 3D structure predictions of all the monomers and homotrimers. Therefore, in our 3D models, the N-terminal regions were not merged with the rest of the structure.

The results of the structural homology search using Dali showed that the head-like regions of gp528 (residues 1–395) and gp531 (1–313) contain five and four NTDs, respectively. At its N-terminus, RaK2 gp528 has a triangular domain NTD1 (formed by three anti-parallel β-sheets), which is capped by a triple-helical stem. This whole module showed structural similarity to the C-terminal region (aa 1103–1239) of the bacteriophage T4 long tail fiber protein 34 (5nxh [54]). In T4, the C-terminus of gp34 binds to gp35 and is also important for folding [54]. Interestingly, according to Dali, a triangular domain similar to gp528 NTD1 is also present in the TSP of *Acinetobacter* phage vb_AbaP_AS12 (6eu4) and in *Pseudomonas aeruginosa* R2 pyocin fiber PA0620 (6cl6 [55]) but is found in the C-terminal region in both proteins. In addition to NTD1, AlphaFold2 predicted four CBA120 TSP4-like XD domains (Table S2) in the structure of the gp528 head region, which is separated from the body by an α-helical neck. Of note, according to Dali, the predicted 3D structure of gp528 also includes a fifth TSP4-like XD domain located in the body region (Figure 2B, Table S2). Figure 2 shows that following a long α-helical neck (predicted with poor confidence scores, see Figure S3), the catalytic right-handed parallel β helix domain CD (residues 648–986) and the β-sandwich domain CTD1 (res. 1006–1113) comprise the C-terminal module of RaK2 gp528. According to Dali, gp528 CTD1 is similar to the collagen-binding domain of Clostridium histolyticum class I collagenase (1nqd [56]), suggesting that gp528 CTD1 may promote virion binding to the host cell.

As mentioned above, a 310-aa head region of RaK2 gp531 was predicted to accommodate four NTDs: two that show similarity to the head domains of CBA120 TSPs, and two that are structurally similar to the XD domains of TSP4 (Table S2). The body region of gp531 is dominated by the putative catalytic β-helical domain (residues 402–740), followed by two C-terminal domains. As with gp532 and gp533, Dali returned the poly(beta-D-mannuronate) C5 epimerase 4 (2pyg [57]) from *Azotobacter vinelandii* as the best match to the catalytic domain of gp531, which is also structurally similar to that of the preneck appendage protein phi29 gp12 (3gq9 [52]). Interestingly, gp531 CTD1 was identified by Dali as being close to a glycan-binding-like domain I from *Acinetobacter*-secreted protease CpaA (6o38 [58]), suggesting that CTD1 likely facilitates binding to surface polysaccharides.

**Figure 3:**
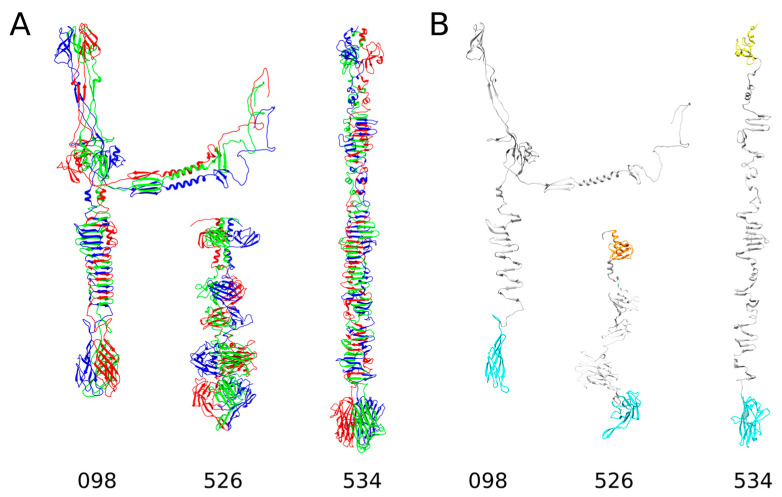
The predicted structures of RaK2 gp098, gp526, and gp534. (**A**) Ribbon representation of predicted trimeric assemblies; the individual monomers are colored in red, green, and blue. (**B**) Ribbon representation of monomers obtained from the predicted 3D structures of trimers. The color code is as follows: orange—domain D1, yellow—D2, gray—central domains with structural homologs in other phage proteins, cyan—CTDs.

As can be seen from the results presented in Table S2 and Figure 3, three RaK2 TFPs (gp098, gp526, and gp534) do not have the predicted polysaccharide-degrading domains. Moreover, while the 3D structure of gp526, as modeled by AlphaFold2, is somewhat similar to that of the RaK2 TFPs discussed above, the structures of gp098 and gp534 are completely different.

According to AlphaFold2, the 110 N-terminal residues of gp526 form a six-stranded beta sandwich preceded by an α-helix, i.e., a fold similar to that of the head domain D1 of CBA120 TSP1 (4oj6 [30]). The body of gp526 (residues 113–432) comprises two triangular domains (of which one, BD1, is similar to a Knob 2 domain of *P. aeruginosa* R1 pyocin fiber PALES_06171; 6cl5 [55]) and a large β-sandwich domain, which was a good match to that of a glycan biofilm-modifying enzyme WceF from *Pantoea stewartii* (6tgf [59]). Another β-sandwich domain is located at the gp526 C-terminus and, according to Dali, its structural homolog is also present in the TSP from *Acinetobacter baumanii* phage phiAB6 (5jse [60]).

The AlphaFold2 prediction of gp534 yielded a rod-shaped trimeric structure (Figure 3) characterized by an elongated stem-like body region and a rounded C-terminal domain. Remarkably, the predicted NTD of gp534 (residues 1–77) was identified by Dali as a structural homolog of the D2 head domain of CBA120 TSP3 (5w6f [29]), whereas the closest match to the gp534 CTD (aa 523–688) was a receptor-binding domain of human ectodysplasin A1 (1rj7 [61]), which has a complex jelly-roll fold [61]. It should be noted that the RBP of *Lactococcus lactis* phage 1358 also has a C-terminal polysaccharide-binding domain of similar structure (4rga [62]), suggesting that RaK2 gp534 is most likely an RBP, even though it has no identifiable depolymerase domains in its 404 aa body region. The latter is made up of mostly three-stranded antiparallel β-sheets connected by unstructured loops of varying length. Similar repetitive structural elements were also found in T4 gp34, gp12, and Mu gp49, and all these proteins require a chaperone for correct assembly of trimers [63,64].

Finally, as shown in Figure 3 and Figure S4, AlphaFold2 failed to predict a high-quality monomer or trimer structure of the full-length RaK2 gp098. There were several possible reasons for this: (i) the lack of elucidated structures of similar sequence, (ii) gp098 folds into the correct 3D structure only after contact with an unidentified partner protein, and (iii) gp098 functions as a multimer but not a trimer. Whatever the case, Dali reported high-confidence functionally relevant matches only to the modeled C-terminal region (residues 370–585) of gp098. This region (Figure 3) starts with a CTD1 domain (antiparallel five-stranded β-sheet) followed by a motif of parallel β-strands forming a triple β-helix in the homotrimer and ends with a β-sandwich CTD2 domain. According to Dali, gp098 CTD1-β-helix module has a structural homolog in the phage T4 long tail fiber protein 34 (region corresponding to the P3 domain and following β-helix; [54]), while CTD2 is a good match to the receptor-binding C-terminal domain of *P. aeruginosa* R2 pyocin fiber PA0620 [55], suggesting that gp098 is an RBP as well. It should also be noted that both T4 gp34 and PA0620 function as trimers [54,55].

To summarize, bioinformatic analysis showed that, even though they contained domains commonly found in phage tail proteins, all the predicted RaK2 TFPs were remarkably different from the known TFPs or TSPs. The case of gp098 was particularly interesting. Given the location of its gene and the results of the in silico analysis, the question arose whether this protein is a structural component of RaK2 long tail fibers. Thus, to clarify this and to determine the structure-function relationships of other RaK2 TFPs, we then aimed to produce recombinant soluble proteins.

### 2.2. Production of Recombinant RaK2 TFPs

As discussed in the previous section, domains essential for host recognition and polysaccharide degradation are located close to the C-terminus or, in some cases, at the C-terminus of the RaK2 TFPs; therefore, to avoid interfering with these functions, the N-terminal tags for protein purification were introduced through cloning of individual genes or gene fragments.

For the optimal production of individual predicted RaK2 TFPs, we investigated different expression strains (*E. coli* BL21(DE3), HMS174, Rosetta, Arctic Express, ER2566), plasmid vectors (pET-16b, pET-28b, pGEX-2T), and various expression conditions (IPTG concentration, 0.1–1.0 mM; temperature, 16–37 °C; induction time, 2–24 h). However, despite numerous strategies, only five RaK2 TFPs, gp526, gp529, and gp531–gp533, were successfully expressed as soluble N-His-tagged proteins. As seen in Table 1, most of the recombinant RaK2 TFPs were obtained using *E. coli* Rosetta (DE3) cells, and only 10xHis_gp532 was produced in HMS174. Moreover, prolonged induction at lower temperatures was needed in most cases, except for 10xHis_gp533, which was induced at 30 °C.

Although many recommendations on improving recombinant protein production and minimizing inclusion body formation may be found in the literature [65,66,67,68], the expression of individual TFPs, notwithstanding exhaustive efforts to optimize the expression conditions, is often unsuccessful [23,64,69,70]. In such cases, truncated protein variants are usually generated [23,71,72,73]. Since we could not obtain the full-length recombinant gp098, gp527, gp528, gp530, and gp534, individual genes fragments were cloned. However, though we tested the constructs of various lengths and fusions, we obtained only the C-terminus of gp098 (aa 368–595) and the N-terminal part of gp528 (aa 1–372) in sufficient quantities in a soluble form (Table 1).

**Figure 4:**
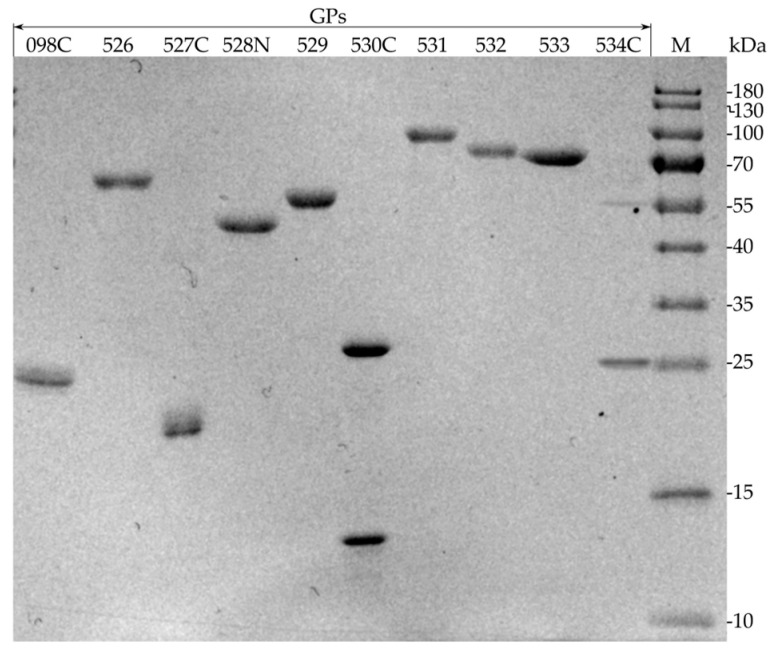
SDS-PAGE analysis of purified recombinant RaK2 TFPs (~1–5 µg). M—prestained protein ladder (#26616), 10–180 kDa (Thermo Fisher Scientific, Vilnius, Lithuania).

For gp527, gp530 and gp534, only expression of the truncated variants yielded sufficient quantities of protein, but solubility was not affected by different expression conditions. For this reason, these three recombinant proteins were purified under denaturing conditions (Figure 4), as described in the Section 4. It is important to note that all three recombinant proteins showed a propensity to aggregate after urea removal by dialysis.

### 2.3. Depolymerase Activity Test

As discussed above, bacteriophage RaK2 encodes at least ten TFPs, seven of which are reliably predicted to contain a putative polysaccharide-degrading domain. However, it has been noted previously that, in the case of bacteriophages with multiple depolymerases, a single TFP usually confers specificity for a single capsular serotype [19,27,37]. Based on this, we hypothesized that only one RaK2 TFP is likely responsible for the recognition and initial attachment of its host, *K. pneumoniae* KV-3 cells.

**Figure 5:**
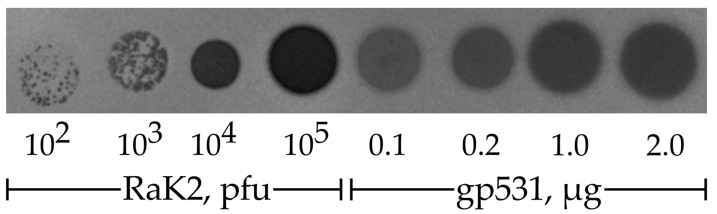
Capsule-degrading depolymerase activity of RaK2 gp531. A spot test was performed on the lawn of *K. pneumoniae* KV-3; CsCl-purified RaK2 phage suspension was used as a control.

To determine which of the putative RaK2 depolymerases could recognize and degrade the capsule of KV3, purified full-length recombinant proteins gp526, gp529, and gp531–533 were used in a spot test. Of note, recombinant gp526, which has no predicted polysaccharide-degrading domains, was also included as a negative control. The spot test results revealed that out of the four RaK2 TFPs, only gp531 was capable of forming a plaque-like translucent spot on the lawn of *K. pneumoniae* KV-3 (Figure 5).

The recombinant RaK2 TFPs were then assayed on individual lawns of four other *Klebsiella* strains (namely, *Klebsiella* sp. KV-1, *K. oxytoca* ATCC 8724, *K. pneumoniae* ATCC BAA-1705, and *K. pneumoniae* 279) [35] known to be resistant to RaK2 infection. As expected, we detected no depolymerase activity.

Taken together, depolymerase activity test results indicated that during RaK2 adsorption on *K. pneumoniae KV-3* cells, gp531 likely acts as a primary RBP that specifically recognizes and cleaves the capsule of this particular host.

### 2.4. Western Blot Analysis of Antisera Raised against Recombinant RaK2 TFPs

**Figure 6:**
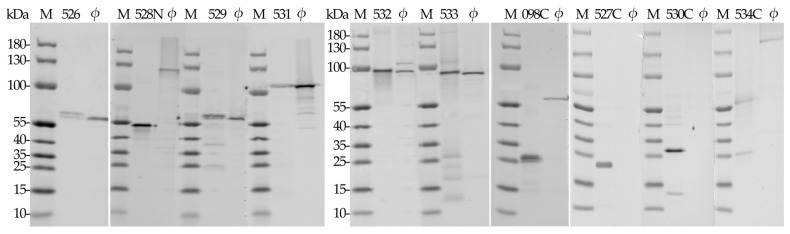
Western blot analysis of antisera raised against individual recombinant RaK2 TFPs. Target recombinant proteins (~0.1–31 ng) are indicated at the top; ϕ—CsCl-purified RaK2 virion samples (PFU, ~1.5–4.6 × 10^8^). M—Prestained protein ladder (#26616), 10–180 kDa (Thermo Fisher Scientific, Vilnius, Lithuania).

To investigate the role of individual RaK2 TFPs in phage infectivity, polyclonal antibodies were raised against each of the ten recombinant proteins. Then, Western blot analysis was performed to investigate the ability of anti-gp098C, anti-gp526, anti-gp527C, anti-gp528N, anti-gp529, anti-gp530C, anti-gp531, anti-gp532, anti-gp533, and anti-gp534C sera to detect the respective recombinant and native RaK2 TFPs separated by SDS-PAGE (Figure 6).

As seen in Figure 6, Western blotting revealed that seven out of ten antisera show reactivity to both recombinant and native RaK2 TFPs. However, traces of cross-reactivity were observed for anti-gp531 and anti-gp532. The product of RaK2 g531 shared several regions of sequence homology with gp528 (aa 85–238, 163–327, and 233–354), gp532 (aa 17–322), gp533 (aa 5–234 and 249–369), and gp534 (aa 5–89). Thus, the additional bands observed for anti-gp531 and anti-gp532 sera were likely due to the slight cross-reactivity of these antibodies with several other TFPs.

Figure 6 shows that in Western blot analysis anti-gp527C and anti-gp530C sera did not appear to interact (or interacted very weakly, well beyond the limits of detection) with RaK2 virion proteins separated by denaturing gel electrophoresis. Nevertheless, as shown in Section 2.6, both of these antisera were able to bind to non-denatured native TFPs present in the RaK2 virion structure, indicating that anti-gp527C and anti-gp530C sera contain predominantly conformation-specific [74] antibodies. Interestingly, Vinga and colleagues [71] reported similar results for an antiserum raised against an insoluble C-terminal fragment of the phage SPP1 tail protein gp21.

Of particular interest were the results of the Western blot analysis of the anti-gp534C serum. Figure 6 shows two protein bands in lane 534C. The lower band corresponds to recombinant gp534C, with a predicted molecular weight of 28 kDa (Table 1). Remarkably, the molecular weight of the upper ~57 kDa protein band is very similar to that predicted for the gp534C dimer, i.e., 56.8 kDa. Similar to IgG raised against gp527C and gp530C, anti-gp534C serum did not seem to interact with denatured native RaK2 gp534, which has a theoretical molecular weight of ~71 kDa. Instead, as shown in Figure 6, this antiserum interacted with an unidentified ~150 kDa RaK2 structural protein. Interestingly, immuno-gold TEM analysis (Section 2.6) showed that antibodies raised against gp534C specifically recognize and bind the tips of the long tail fibers of this phage. Although a protein of this size is not present in the structure of RaK2 LTFs, coincidentally, the theoretical mass of gp534 dimer is 142 kDa. Thus, although additional detailed studies are needed to fully elucidate this phenomenon, the results of the Western blot analysis suggested that the oligomers formed by RaK2 gp534 are, at least partially, resistant to reducing conditions, and that the C-terminus of the protein retains this property.

### 2.5. RaK2 Inhibition with Antisera

**Figure 7:**
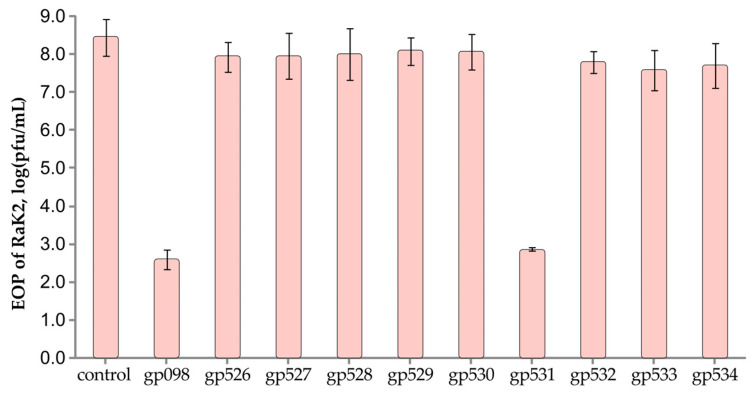
Effect of antibodies raised against individual TFPs on the EOP of RaK2. Data represented as mean ± SD (n = 4). The statistical significance was calculated using Student’s *t* test (*p* < 0.001).

We then investigated the effect of polyclonal antibodies raised against individual recombinant TFPs on RaK2 infection (Figure 7). The idea behind this test is that if specific antibodies block the phage receptor-binding protein, a significant reduction in the efficiency of RaK2 plating should be observed.

As seen from the results presented in Figure 7, pre-treatment of the phage particles with anti-gp531 serum resulted in a ~100,000-fold reduction in RaK2 plating efficiency (EOP), which was further evidence that this protein functions as a primary RBP during adsorption on *K. pneumoniae KV-3* cells. Whereas no significant difference in RaK2 infectivity was observed after pre-incubation with antibodies raised against TFPs gp526–gp530 and gp532–gp534, the EOP of RaK2 was significantly reduced after pre-incubation with anti-gp098C (Figure 7), indicating that this protein is also essential for KV-3 infection.

### 2.6. Adsorption Inhibition Assay

**Figure 8:**
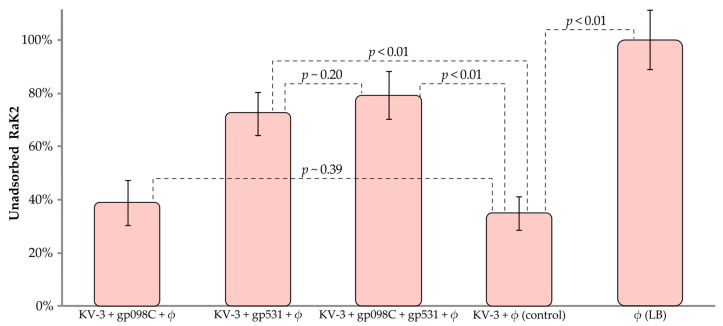
Inhibition of RaK2 adsorption by gp531 and gp098. Error bars indicate standard deviations (SDs). In total, six biological replicates were conducted. ϕ—CsCl-purified RaK2 phage suspension (PFU, ~1.5–4.6 × 10^8^).

To confirm that bacteriophage RaK2 uses the capsule of KV-3 as a primary receptor, an adsorption inhibition assay was performed where we examined the ability of the phage particles to bind to KV-3 cells treated with gp531, gp098, or a combination of both (Figure 8).

Figure 8 shows that the efficiency of RaK2 adsorption to gp531-treated KV3 cells was significantly reduced, indicating that both the phage and the TFP target the same moieties of the host cell surface. In contrast, pre-incubation of *K. pneumoniae* KV-3 cells with gp098C had no significant effect on RaK2 adsorption. Furthermore, no statistically significant increase in free phage titer was observed when bacteria were treated with both gp531 and gp098. These results suggested one of two possibilities: either gp098 is not an RBP, or it is a secondary RBP, which requires the coordinated action of gp531 to reach its receptor. The results of the bioinformatic analysis seemed to support the latter hypothesis, but they also clearly implied that gp098 is unlikely to be a long tail fiber protein. Therefore, to clarify this and to explore the location of each TFP in the RaK2 phage particle, we performed the immunogold-TEM analysis.

### 2.7. AFM and Immunogold-TEM Analyses

**Figure 9:**
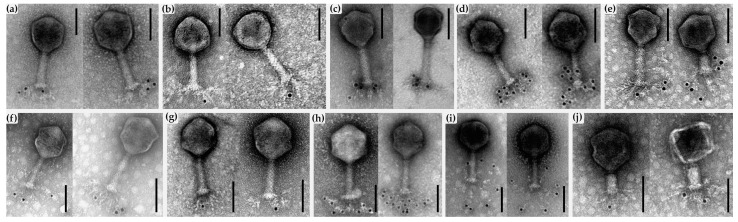
TEM analysis of immunogold-labeled RaK2 phage particles. The primary antibodies are indicated; black dots represent secondary gold-conjugated IgG. (**a**) gp526, (**b**) gp527, (**c**) gp528, (**d**) gp529, (**e**) gp530, (**f**) gp531, (**g**) gp532, (**h**) gp533, (**i**) gp534, (**j**) gp098. Scale bars indicate 100 nm.

Immunogold-TEM analysis (Figure 9) was conducted by using antibodies raised against the recombinant RaK2 TFPs, together with secondary gold-conjugated anti-rabbit IgG (Sigma-Aldrich, Wicklow, Ireland). This analysis allowed us to confirm the presence of all ten proteins in the virion structure and to establish their approximate location in the virion.

Figure 9 shows that gp526–534 are located in the long tail fibers of bacteriophage RaK2. In the case of anti-gp528N serum (Figure 9c), labeling was mainly observed near the baseplate, in the proximal regions of the LTFs. This was consistent with the results of the bioinformatics analysis, which indicated that the N-terminal region of gp528 is responsible for LTF attachment to the baseplate.

Based on the results of immunogold-TEM analysis, gp526, gp527, and gp532 occupy the middle region of the LTFs, while gp530 and gp531 are mostly found at the distal parts. The localization of gp533 and gp529 is unclear owing to the high labeling density, but it was evident that in both cases the gold particles are located closer to the distal end of the RaK2 LTFs.

**Figure 10:**
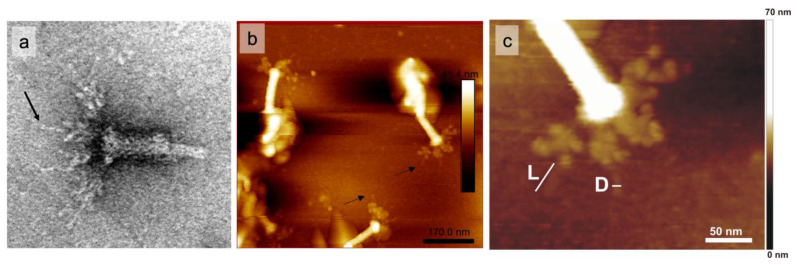
TEM (**a**) and AFM (**b**,**c**) imaging of CsCl-purified RaK2 phage particles. Black arrows indicate the distal rod-shaped protrusions. L—protrusion length (31.85 ± 5.06 nm, n = 68), D—protrusion diameter (2.0 ± 0.27 nm, n = 8).

In the case of anti-gp534C serum (Figure 9i), the labeling was observed exclusively at the very tips of the RaK2 LTFs. As discussed in Section 2.1, AlphaFold2 prediction of gp534 trimer yielded an elongated rod-shaped structure of ~37.6 nm in length and ~2.4 nm in diameter (as measured using UCSF Chimera [75]), with a rounded carbohydrate-binding domain (of ~4.5 nm in diameter) at the C-terminus. Interestingly, in some TEM micrographs of the RaK2 phage, we could indeed observe the distal rod-shaped structures in its LTFs (Figure 10a). To verify this and better understand the morphology of RaK2 LTFs, we analyzed CsCl-purified phage particles by atomic force microscopy (AFM).

Figure 10b shows that AFM imaging confirmed the presence of a protruding distal rod-shaped structure in the RaK2 LTFs. In addition, the length and diameter of these distal protrusions (31.85 ± 5.06 nm and 2.0 ± 0.27 nm, respectively) were in reasonably good agreement with the values obtained from the 3D model of gp534 trimer.

Finally, as can be seen in Figure 9j, the polyclonal antibodies raised against gp098C predominantly labeled phage particles with contracted tails, indicating that the antigen is likely unavailable in virions with non-contracted tails. Since LTFs of all virion states are readily accessible to antibodies, the latter result, together with the data obtained from structure modeling, suggested that gp098 is not a component of RaK2 long tail fibers but rather a baseplate protein.

## 3. Discussion

Bacterial viruses with genomes larger than 200 kb are called jumbo phages [76]. Because of their genome size, such viruses can and often do encode unique and complex structural elements rarely observed in smaller phages. A good example of jumbo viruses is *Klebsiella* phage RaK2, whose ~346 kb genome encodes probably the most elaborate long tail fibers found in phages [19,35,77]. To date, only the *Klebsiella*-infecting virus phiK64-1 has been shown to have a seemingly identical virion morphology [36]. According to the latest ICTV taxonomy release, the two viruses, whose unique tail fibers have not been studied thus far, now form the newly created genus *Alcyoneusvirus* [38]. Therefore, in this study, we combined an in silico approach with biological and in vitro experiments to shed light on the architecture of the alcyoneusvirus adsorption apparatus by studying the putative TFPs of bacteriophage RaK2.

**Figure 11:**
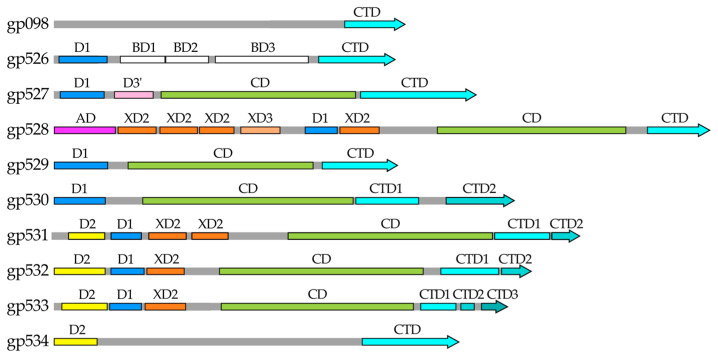
Schematic representation of the domain architecture of RaK2 TFPs.

Our results clearly indicated that of the ten RaK2 TFPs investigated during this study, seven have a depolymerase domain (Figure 11). Yet, despite the large number of polysaccharide-degrading RBPs, RaK2 is known to infect a single host, *K. pneumoniae* veterinary isolate KV-3.

For successful adsorption on *K. pneumoniae* KV-3 cells, two RaK2 proteins, gp531 and gp098, are absolutely essential. The product of RaK2 g*531* is a primary RBP, which specifically recognizes and cleaves the capsule of KV-3. Meanwhile the function of gp098 in RaK2 adsorption is unclear. Bioinformatics analysis indicated that gp098 is a receptor-binding protein whereas immunogold-TEM analysis showed that it is located at the bottom of RaK2 baseplate. As discussed in the Introduction section, in the case of the much-studied *E. coli* myophage T4, the six short tail fibers (formed by the trimers of gp12) are located at the bottom of the baseplate [5,10,11]. In free T4 particles, the STFs are folded (so that the C-termini of gp12 are simultaneously bound to their amino-terminal parts and gp10) and unfold only after reversible attachment [5,10,11,78]. Coincidently, since we could not obtain the full-length recombinant gp098, N-his-tagged C-terminus of this protein was produced. Nevertheless, as discussed earlier, not all tailed phages use two sets of tail fibers for adsorption. Many viruses initially bind to the host cell via the tail fibers but achieve an irreversible binding and subsequent genome transfer through the contact of different baseplate structures (e.g., the tail tip or the central fiber) with the cell surface [5,10]. Since no RaK2 short tail fibers were observed in our study, we propose that gp098 is a baseplate protein that likely acts as a secondary RBP and is involved in irreversible adsorption. To reach its receptor, possibly hidden by the KV-3 capsule, gp098 requires the coordinated action of the long tail fiber protein gp531, for both capsule removal and correct positioning, and/or signal transduction after initial attachment. Notably, according to the data given in Table S1, RaK2 gp098 and phiK64-1 S2-8 are 100% identical, highlighting the importance of this protein in the infection process of alcyoneusviruses.

Based on our results, the long tail fibers of RaK2 comprise nine TFPs (gp526–gp534) that contain conserved N-terminal domains necessary for the formation of these structures (Figure 11). All of these domains are structurally similar to those of the CBA120 TSPs. As discussed above, *E. coli* phage CBA120 has four TSPs [27]. The main organizer of the TSP1-4 complex is TSP4, the largest of the four proteins with the longest N-terminal region (Figure S1). This region contains the N-terminal triple β-helix domain AD, which is required for the complex to attach to the virion [27,32]. Our results indicated that in RaK2 the primary TFP initiating the assembly of the tail fiber network is gp528, which may be considered an ortholog of CBA120 TSP4. Figure 11 shows that RaK2 gp528 has an N-terminal 105 aa anchor, which is likely responsible for the attachment of the whole tail fiber to the baseplate. According to Dali, the anchor domain of gp528 showed no homology to that of CBA120 TSP4, which is to be expected since both proteins interact with genetically distant baseplates. Notably, BlastP analysis indicated that at the amino acid sequence level, gp528 AD is highly conserved among alcyoneusviruses but has no homologs in other phages, which likely attests to the uniqueness of this virus group. CBA120 TSP4 has three N-terminal T4 gp10-like domains, two of which, XD2 and XD3, serve as a platform for the binding of other TSPs [32]. The long tail fibers of RaK2 are formed by more than twice as many proteins as the CBA TSP1-TSP4 complex; therefore, unsurprisingly, AlphaFold2 predicted five such domains in gp528. For most of the predicted gp528 XDs, Dali returned TSP4 XD2 as the best match yet found no similarity to T4 gp10, whereas according to HHpred analysis, all gp528 XD domains were homologous to T4 gp10 XD3 (Table S2). Since TSP4 XD2 and T4 gp10 XD3 are involved in protein–protein interactions [11,27,32], it can be assumed that all five XD domains of gp528 are engaged in tail fiber branching. However, both T4 gp10 and CBA120 TSP4 have an N-terminal XD1 domain, which in the case of both proteins, does not interact with any partners and is probably required for overall structure maintenance and spatial separation of downstream domains from AD [11,32]. Therefore, we suggest that the first XD domain of RaK2 gp528 does not participate in protein–protein interactions in order to maintain the mobility of the whole structure. Moreover, as shown in Figure 11, three other RaK2 TFPs have T4 gp10-like domains for branching: two predicted for gp531 and one each for gp532 and gp533. Thus, eight XD domains are sufficient to assemble nine RaK2 TFPs, provided that gp531–gp533 interact with gp528 via their head domains rather than their XDs. Given that the organization of the N-terminal domains of RaK2 gp531–gp533 (Figure 11) is very different from that of CBA120 TSP4 and TSP2 (Figure S1), this mode of interaction is highly likely.

**Figure 12:**
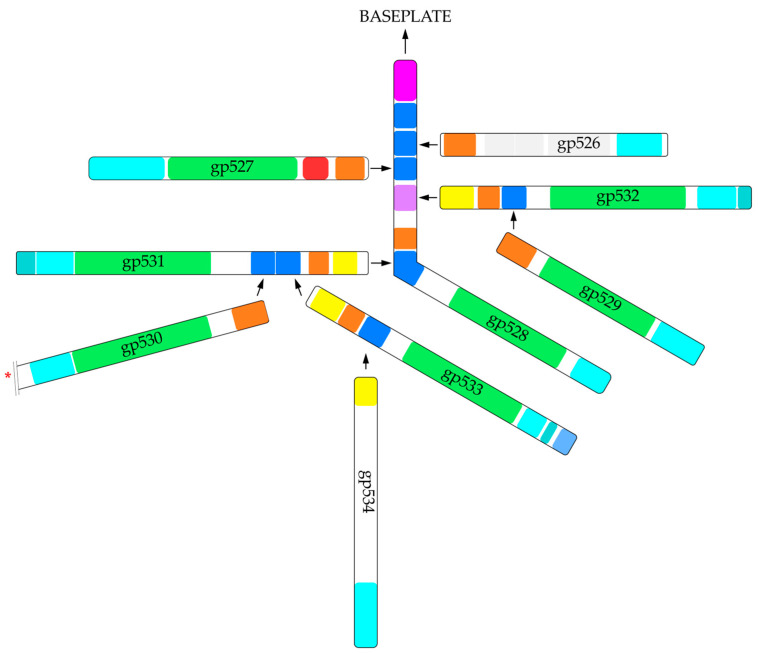
Hypothetical model for the assembly of RaK2 long tail fibers. The color code is as in Figure 11. The red asterisk marks the C-terminus of gp530 after removal of the intra-molecular chaperone. The color code is as follows: pink—anchor domain (AD), orange—D1, yellow—D2, blue—XD2, purple—XD3, green—the catalytic domain, cyan and light blue—CTDs, gray—central domains with structural homologs in other phage proteins.

Given all these insights and based on the results obtained during this study, we propose a tentative hypothetical model for RaK2 long tail fiber assembly (Figure 12). In our model, RaK2 LTFs comprise nine proteins: gp528 directly binds gp531, gp532, gp533, and at least one other TFP, while gp531–gp533 serve as a docking platform for the remaining four RaK2 TFPs. All six RaK2 LTFs, each containing seven polysaccharide-degrading depolymerases, are connected to the baseplate via the N-terminus of gp528.

Finally, as shown in Table S1, the N-terminal domains are highly conserved in both RaK2 and phiK64-1 TFPs, suggesting that these two phylogenetically related viruses likely use a similar mode for long tail fiber assembly. Thus, although further detailed studies are needed, the results presented here provide the first glimpse into the architecture of the *Alcyoneusvirus* host recognition apparatus.

## 4. Materials and Methods

### 4.1. Bioinformatics Analysis

Structures of gp526–gp534 were modeled using a local installation of Alphafold2 (https://github.com/kalininalab/alphafold_non_docker, accessed on 5 November 2022.) [42], obtained with a reduced dataset. In all cases, the number of recycles was increased to 12 while the number of templates to use was increased to 16. To model the trimers, owing to memory limitations, each protein sequence was split into smaller overlapping sections of ~350 residues and modeled separately. The resulting trimeric complexes were then assembled by carefully aligning the overlapping regions using UCSF Chimera v1.16 [75]. All figures showing predicted 3D protein structures were prepared using UCSF Chimera v1.16 [75].

To identify the closest homologs in the PDB database, the predicted domain structures were then submitted to the Dali server, and their corresponding sequences were analyzed using HHpred.

### 4.2. Phage, Bacterial Strains, and Growth Conditions

Bacteriophage RaK2 and its host *K. pneumoniae KV-3* were isolated as described previously. For all phage experiments, CsCl-purified phage stock with a titer of 10^9^–10^11^ pfu/mL was used.

*Escherichia coli* strain DH10B (Thermo Fisher Scientific, Vilnius, Lithuania) was used for plasmid propagation, whereas strains BL21 DE3 (Novagene, Madison, WI, USA), Rosetta DE3 (Novagene, Madison, WI, USA), HMS147 DE3 (Novagene, Madison, WI, USA), Arctic Express DE3 (Agilent Technologies, Santa Clara, CA, USA), and ER2566 (New England Biolabs, Ipswich, MA, USA) were used for the production of recombinant proteins. All bacterial cultures were propagated in Luria–Bertani (LB) medium at 37 °C, unless stated otherwise.

### 4.3. Plasmid Construction

Specific plasmids and primers (Metabion GmbH, Steinkirchen, Germany) used for the cloning of target genes are listed in Table S3. The recombinant plasmids were constructed using standard cloning methods. Briefly, putative tail fiber genes (RaK2 *526*, *529*, *531*, *532*, and *533*) or their fragments (*098C*, *527C*, *528N*, *530C*, and *534C*) were amplified by PCR, using Pfu DNA polymerase (Thermo Fisher Scientific, Lithuania) and CsCl-purified RaK2 suspension as a template. After restriction enzyme digestion, the PCR fragments were cloned into the corresponding pET28b (*098C*, *526*, *527C*, *529*, *530C*, *534C*) or pET16b (*528N*, *531*, *532*, and *533*) expression vectors (Table S3), and the recombinant plasmids obtained were then transformed into *E. coli* DH10B. The plasmid DNA was extracted using the GeneJET Plasmid Miniprep Kit (Thermo Fisher Scientific, Lithuania) from individual colonies selected by PCR, and sequenced (Macrogen, Seoul, Republic of Korea).

### 4.4. Protein Production and Purification

For protein production, the recombinant plasmids were transformed into *E. coli* Rosetta DE3 and the cells were grown in plastic flasks with 50–200 mL Luria–Bertani (LB) medium supplemented with 50 µg/mL ampicillin for pET16b or 35 µg/mL kanamycin for pET28b constructs. At optical density (OD600) of 0.5–0.8, the cultures were cooled on ice for 10 min, and the protein synthesis was induced with 0.5–1.0 mM IPTG (Table 1). The cultures were then incubated at 17–30 °C, with shaking, for 18–21 h (Table 1). The biomass was collected by centrifugation at 2600× *g* for 20 min at 4 °C, resuspended in binding buffer (20–50 mM Tris or sodium phosphate, pH 8.0) supplemented with 1.0 mM PMSF, and disrupted on ice by sonication (25 s × 35 s, up to 10 min) using ultrasound homogenizer (Branson Ultrasonics™ Sonifier™ SFX250, Thermo Fisher Scientific, Waltham, MA, USA).

In the case of soluble His-tagged proteins, the lysates were cleared by centrifugation at 8000× *g*, at 4 °C for 30 min and loaded onto a 1 mL HisTrap nickel chelating column (Cytiva, Marlborough, MA, USA) housed in an AKTA Purifier 100 FPLC system (GE Healthcare, Chicago, IL, USA). Proteins were eluted by 0.5–0.7 M imidazole linear gradient, collecting 0.5 mL fractions. Collected fractions were then analyzed by SDS-PAGE, and those of the greatest purity were pooled, transferred to dialysis bags (12 kDa cut-off, Carl Roth GmbH, Karlsruhe, Germany), and dialyzed overnight against 50 mM Tris (pH 8.0) or 20 mM sodium phosphate (pH 8.0) buffer, at 4 °C with constant agitation. Dialyzed recombinant proteins were concentrated approximately to 1 mg/mL using carboxymethylcellulose. Protein concentrations were determined by Lowry method.

To purify insoluble recombinant proteins gp527C, gp530C, and gp534C, after cell disruption the pellet was collected and dissolved in 6 M urea. The proteins were then purified on HisTrap 1 mL columns according to the procedure described above, except that the protein elution buffer was supplemented with 6 M urea.

### 4.5. Depolymerase Activity Assay

A spot test on the lawns of *K. pneumoniae*. KV-3 was performed to assess the capsule-degrading activity of the recombinant RaK2 proteins following a procedure described previously [79]. Briefly, bacteria were grown in LB medium at 37 °C (450 RPM) till OD_600_ reached 0.4–0.5. Then, 0.5 mL of culture was plated using a standard double-layer agar method and the plates were air-dried (for 30–60 min). Purified recombinant proteins were diluted (from 0.1 to 2.0 μg/mL) with 0.2 M sodium phosphate buffer (pH 6), and 10 μL of the appropriate dilution was spotted on the top of soft agar. Serially diluted RaK2 suspension (10^2^–10^5^ PFU/mL, diluted with LB) was used as a control. The plates were then incubated at 37 °C overnight.

### 4.6. RaK2 Adsorption Inhibition with Recombinant Proteins

*K. pneumoniae KV-3* cells (grown in LB medium at 37 °C, 450 RPM, till OD_600_ reached 1.0) were collected by centrifugation for 2.5 min, 15,200× *g*, at 20–25 °C. The biomass was then washed with 0.2 M sodium phosphate solution (pH 6) and sterile water and resuspended in 20 mM sodium phosphate (pH 6) buffer. Then, ~10 μg gp531, ~15 μg gp098C, a mixture of both proteins, or buffer solution (20 mM sodium phosphate, pH 6.0) as a control was added, and the samples were incubated for 30 min at 30 °C, 450 RPM. After pre-treatment, cells were washed and diluted with LB medium and infected with RaK2 phage for 8 min at 0.5–1.0 MOI. Supernatant was then collected after centrifugation (20 min, 2700× *g*, 4 °C) and used to determine the titer of unadsorbed phage.

### 4.7. Antisera Production

Rabbit polyclonal antibodies against the purified recombinant proteins were generated at the State Research Institute Center for Innovative Medicine in adherence to their ethics-approved protocols. The polyclonal antisera were raised by silver rabbit immunization with 4 × 0.25 mg of each protein per rabbit. Each 1 mL dose, prepared in 50 mM Tris supplemented with glycerol (10%) pH 8.0, was stored at −20 °C before use. Immunization was performed in 2-week intervals with thawed antigen portion emulsified by 1:1 volume ratio with either Freund’s complete (for the 1st immunization) or incomplete adjuvant (for 2nd–4th immunizations). Rabbits were injected subcutaneously with 8 × 0.2 mL of the corresponding antigen dose by insulin syringe, and 2 × 0.2 mL portions were injected into muscle tissue. Two weeks after the last immunization, rabbit blood was collected and the antisera were extracted by centrifugation (1500× *g*, 20 min). The antisera were then stored at −80 °C before use [80].

### 4.8. RaK2 Adsorption Inhibition with Policlonal Antiserum

Purified RaK2 suspension (~10^10^ pfu/mL) was incubated with the corresponding antiserum diluted 1:100 in 137 mM NaCl, 2.7 mM KCl, 8 mM Na_2_HPO_4_, and 2 mM KH_2_PO_4_, pH 7.4 at room temperature for 1 h with gentle agitation. Then, the determination of the efficiency of plating was performed as described previously [81].

### 4.9. Immunogold Labeling and TEM Analysis

For immunogold labeling, purified RaK2 suspension (~10^10^ pfu/mL) was incubated for 2 min at room temperature individually with each antiserum (*v*:*v*, 1:1) diluted up to 1:100 in PBS (20 mM sodium phosphate, 0.15 M NaCl, pH 8.1). Then, the nickel-copper grids were placed on a drop of the phage-antiserum solution and incubated for 15–25 min at 37 °C. The grids were washed by transferring them onto five consecutive drops of PBS for 1 min at room temperature, and then the sample-coated grids were placed on a drop of the goat anti-rabbit immunoglobulin G 10-nm gold conjugate solution (Sigma-Aldrich, St. Louis, MO, USA) diluted up to 1:100 in PBS, and incubated at 37 °C for 15–25 min. The grids were then washed as described above and stained with 2% uranyl acetate for 1–1.5 min at 20–25 °C. Air-dried grids were analyzed with a Morgagni 268D transmission electron microscope (FEI, Hillsboro, OR, USA).

### 4.10. AFM Analysis

The bacteriophage sample (1.6 × 10^10^ pfu/mL) was prepared in 20 mM sodium phosphate buffer (pH 8.1) with 150 mM NaCl. Then, 10 μL of the phage sample solution was spread on 0.01% poly-L-lysine-modified mica (freshly cut mica sheets (IV grade, SPI Supplies. Inc., West Chester, PA, USA) incubated in 0.01% poly-L-lysine solution for 30 min). After 2 min of incubation, the samples were washed with MilliQ water (18.2 MΩ·cm), dried under nitrogen flow, and visualized by the atomic force microscope system DimensioIcon (Bruker, Billerica, MA, USA) under constant amplitude in the tapping mode in the air with TESPA cantilevers (Bruker, Billerica, MA, USA), probes with a nominal spring constant of 2–40 N/m. The image analysis was performed with NanoscopeAnalysis (1.9) software and WSxM (5.0) packages.

## Figures and Tables

**Table 1:** Conditions for the production of recombinant RaK2 TFPs.

Recombinant Protein	MW, kDa ^1^	MW, kDa ^2^	IPTG, mM	Temperature, °C	Time, h	*E. coli* Strain
098C	62.9	26.3 *	1.0	22	18	Rosetta DE3
526	63.1	66.1	1.0	20	18	Rosetta DE3
527C	79.5	21.6 *	0.5	30	18	Rosetta DE3
528N	121.8	46.3 *	0.8	25	18	HMS174 DE3
529	62.5	64.9	0.5	17	17	Rosetta DE3
530C	86.1	29.7 *	0.5	30	18	Rosetta DE3
531	97.8	100.3	0.5	17	19	Rosetta DE3
532	87.3	89.8	1.0	18	17	HMS174 DE3
533	82.8	85.4	0.5	30	21	Rosetta DE3
534C	70.8	28.4 *	0.5	30	18	Rosetta DE3

^1^ MW of the native protein, calculated from the predicted amino acid sequence. ^2^ MW of the recombinant protein, calculated from the predicted amino acid sequence. * indicates truncated variants. Recombinant proteins purified under denaturing conditions are highlighted in gray.

## Data Availability

All data underlying the results are available as part of the article and no additional source data are required.

## Supplementary Materials

The supporting information can be downloaded at: https://www.mdpi.com/article/10.3390/ijms24119320/s1.
