# Supplementary material for: Insights into the Alcyoneusvirus Adsorption Complex
Source: Int J Mol Sci. 2023 May 26;24(11):9320. doi: 10.3390/ijms24119320 (PMC10253068; doi:10.3390/ijms24119320)
Supplement: Supplementary file 1 [file ijms-24-09320-s001.zip › ijms-2407475-supplementary.pdf]

Supplementary Material for:
International Journal of Molecular Sciences

Insights into the *Alcyoneusvirus* adsorption complex

Algirdas Noreika[1], Rasa Rutkiene[1], Irena Dumalakienė[2], Rita Vilienė[2], Audrius Laurynėnas[3], Simona Povilonienė[1], Martynas Skapas[4], Rolandas Meškys[1] and Laura Kaliniene[1*]

[1] Department of Molecular Microbiology and Biotechnology, Institute of Biochemistry, Life Sciences Center, Vilnius University, Saulėtekio av. 7, LT-10257 Vilnius, Lithuania
[2] Department of Immunology; State Research Institute Center for Innovative Medicine, Santariškių st. 5, LT-08410 Vilnius, Lithuania
[3] Department of Bioanalysis, Institute of Biochemistry, Life Sciences Center, Vilnius University, Saulėtekio av. 7, LT-10257 Vilnius, Lithuania
[4] Department of Characterisation of Materials Structure, Center for Physical Sciences and Technology, Saulėtekio av. 3, LT-10257 Vilnius, Lithuania
* Correspondence: laura.kaliniene@bchi.vu.lt

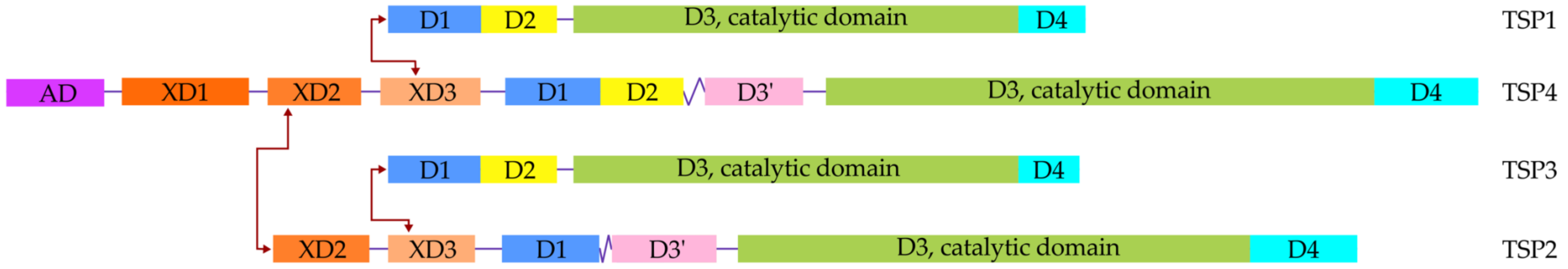

Figure S1. Domain architecture of CBA120 TSPs [27,29-32]. Red arrows indicate protein-protein interactions identified and described in [27] and [32].

Table S1. Relationships between RaK2 TFPs and their K64-1 counterparts, determined using BlastP.

RaK2 TFP	Accession No.	Length aa	Range	Coverage	E-value	Identities (%)	K64-1 Protein	Accession No.	Capsule specificity*
gp098	YP_007007253	595	1-595	100%	0	595/595 (100%)	S2-8	YP_009153165	----
gp526	YP_007007681	580	1-121	20%	5e-37	75/122 (61%)	S1-2	YP_009153195	KN4
gp527	YP_007007682	715	1-688	96%	1e-46	187/718 (26%)	S1-1	YP_009153197	K11
gp528	YP_007007683	1113	1-632	56%	0	518/633 (82%)	S2-1	YP_009153198	KN5
gp529	YP_007007684	584	1-584	100%	0	581/584 (99%)	S2-2	YP_009153199	K25
gp530	YP_007007685	779	1-779	100%	0	754/779 (97%)	S2-3	YP_009153200	K35
-"-	-"-	-"-	5-93	11%	3e-06	30/90 (33%)	S1-3	YP_009153196	K21
gp531	YP_007007686	895	89-347	28%	6e-146	231/259 (89%)	S2-4	YP_009153201	K1
gp532	YP_007007687	806	13-308	36%	2e-143	225/298 (76%)	S2-5	YP_009153202	K64
gp533	YP_007007688	767	1-767	100%	0	690/767 (90%)	S2-6	YP_009153203	K30, K69
gp534	YP_007007689	688	85-668	88%	0	449/605 (74%)	S2-7	YP_009153204	----
-"-	-"-	-"-	1-302	44%	6e-70	151/307 (49%)	-"-	-"-	-"-

*- indicates the specificity of the corresponding purified K64-1 proteins as shown in [36,37];
-"- indicates „same as above".

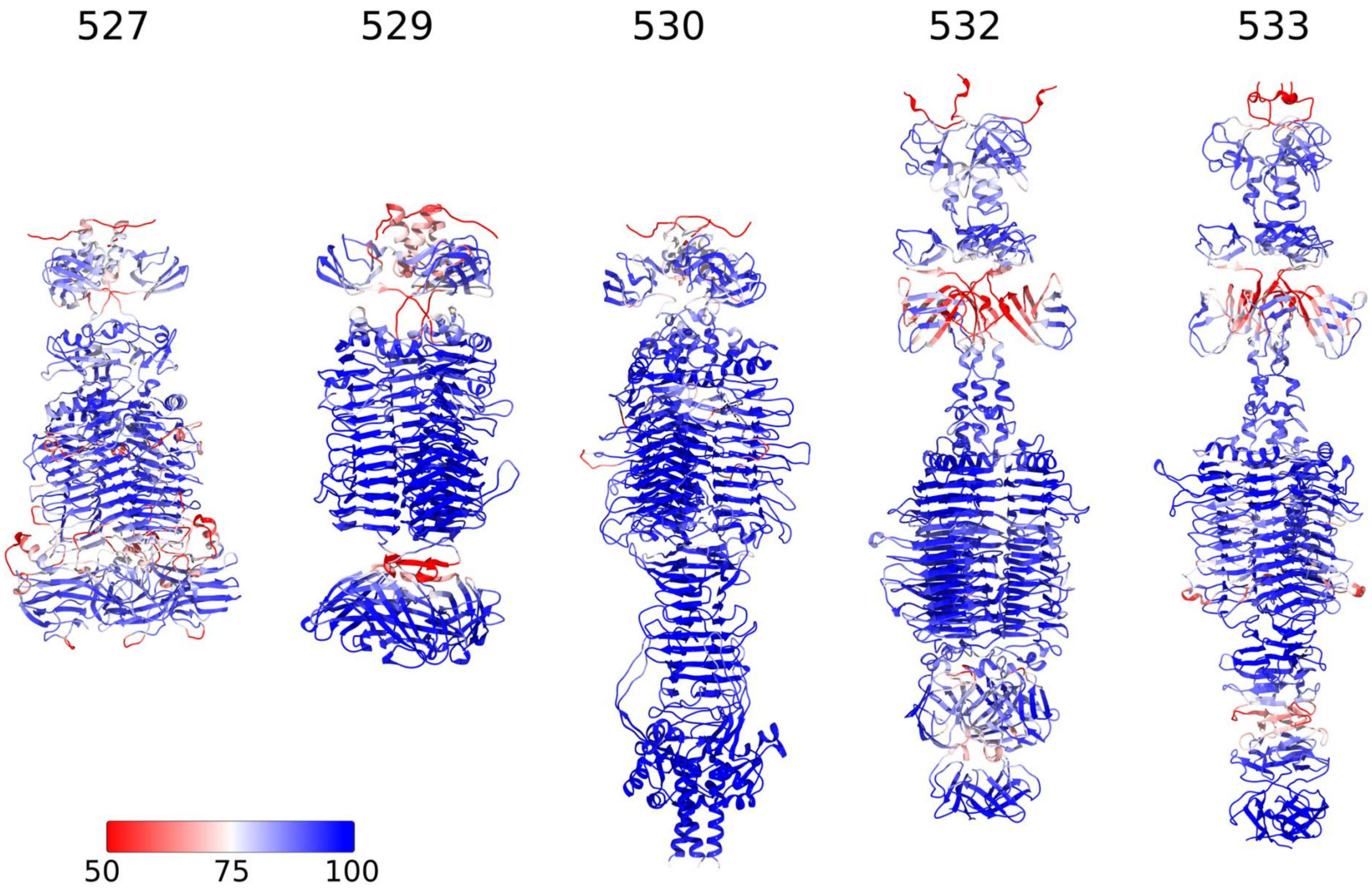

Figure S2. AlphaFold2 predicted trimeric structures of RaK2 gp527, gp529, gp530, gp532, and gp533, colored by the pLDDT value. The color code is at the bottom left.

Table S2. Dali and HHpred analyses of predicted RaK2 TFP domains.

Gp527 (715 aa)				
	Head region		Body region	
	**NTD1** (11-86)	**NTD2** (103-169)	**CD** (182-512)	**CTD** (520-715)
**Dali** top hit**<br>(PDB ID, nres)<br>aligned region (domain) in the matching structure<br>Z-score, RMSD Å/aligned Cα	*E. coli* phage CBA120 **TSP1**<br>(4oj6, 753 aa)<br>1-79 (D1)<br>Z=11.7, Rmsd=1.2 /73	*E. coli* phage CBA120 **TSP2**<br>(5w6p, 676 aa)<br>19-90 (D3′)<br>Z=9.2, Rmsd=1.8/66	*E. coli* phage CBA120 **TSP4**<br>(5w6h, 697 aa)<br>233-551 (D3)<br>Z=25.4, Rmsd=2.5/290	*Rhodothermus marinus* xylanase<br>(2y6h, 167 aa)<br>1-167 (CBM4-2)<br>Z=14.2, Rmsd=2.6/148<br><br>*E. coli* phage CBA120 **TSP4**<br>(5w6h, 697 aa)<br>541-697 (D4)<br>Z=12.1, Rmsd=3.1/149
**HHpred** top hit**<br>(PDB ID, nres)<br>aligned region (domain) in the matching structure<br>Probability%, E-value	*E. coli* phage vB_EcoP_G7C Gp63.1 (4qnl; 859 aa)<br>1-83 (D1)<br>76.84%, 14	*E. coli* phage CBA120 **TSP2**<br>(5w6s, 680 aa)<br>23-100 (D3′)<br>99.17%, 7e-10	*Acinetobacter phage* AP22 gp54<br>(4y9v; 625 aa)<br>56-432 (ED)<br>99.83%, 3.3e-17	*Paenibacillus barcinonensis* Endo-1,4-beta-xylanase C<br>(4xup; 334 aa)<br>203-333<br>98.07%, 5.0e-4<br><br>*E. coli* phage CBA120 **TSP4**<br>(5w6h, 697 aa)<br>590-694 (D4)<br>94.38%, 1.6

Gp529 (584 aa)			
	Head region		Body region
	**NTD1** (1-92)	**CD** (126-440)	**CTD** (455-584)
**Dali** top hit*<br>(PDB ID, nres)<br>aligned region (domain) in the matching structure<br>Z-score, RMSD Å/aligned Cα	*E. coli* phage CBA120 **TSP1**<br>(4oj6, 676 aa)<br>1-79 (D1)<br>Z=11.3, Rmsd=1.6/77	*Azotobacter vinelandii* Poly(beta-D-mannuronate) C5 epimerase 6<br>(5lw3; 381 aa)<br>13-373<br>Z=28.1, Rmsd=2.7/289<br><br>*E. coli* phage CBA120 **TSP2**<br>(5w6s, 676 aa)<br>99-652 (D3)<br>Z=26.9, Rmsd=2.7/288	*E. coli* phage T4 gp9<br>(1qex; 288 aa)<br>171-281 (CTD)<br>Z=9.2, Rmsd=2.9/102
**HHpred** top hit*<br>(PDB ID, nres)<br>aligned region (domain) in the matching structure<br>Probability%, E-value	--------	*Azotobacter vinelandii* Poly(beta-D-mannuronate) C5 epimerase 6<br>(5lw3; 381)<br>16-293<br>99.9%, 8.7e-20<br><br>*E. coli* phage CBA120 **TSP3**<br>(6nw9, 633 aa)<br>215-581 (D3)<br>99.89%, 1.6e-19	------------

Gp530 (779 aa)				
	Head region	Body region		
	**NTD1** (1-88)	**CD** (151-508)	**CTD1** (512-619)	**CTD2** (665-779)

**Dali** top hit**<br>(PDB ID, nres)<br>aligned region (domain) in the matching structure<br>Z-score, RMSD Å/aligned Cα	*E. coli* phage CBA120 **TSP4**<br>(5w6h, 697 aa)<br>1-81 (D1)<br>Z=10.6, Rmsd=1.7/75	*Shigella flexneri* phage Sf6 endorhamnosidase<br>(2vbm, 509 aa)<br>68-393<br>Z=29.7, Rmsd=2.8/285	*E. coli* phage T5 L-shaped TFP<br>(4uw8, 427 aa)<br>183-427<br>Z=14.5, Rmsd=2.6/192	
**HHpred** top hit**<br>(PDB ID, nres)<br>aligned region (domain) in the matching structure<br>Probability%, E-value	*E. coli* phage CBA120 **TSP4**<br>(5w6h, 697 aa)<br>3-83 (D1)<br>94.71%, E=0.13	*Fusarium moniliforme* endopolygalacturonase<br>(1hg8, 349 aa)<br>5-336<br>99.9%, 9.3e-21<br><br>Acinetobacter bacteriophage AP22 TSP gp54<br>(4y9v, 625 aa)<br>56-434<br>99.88%, 3.1e-19	--------	*E. coli* phage T5 L-shaped TFP (4uw8, 427 aa)<br>269-427<br>98.92%, 5.9e-7

**Gp532 (806 aa)**						
	**Head region**			**Body region**		
	**NTD1**<br>(1-88)	**NTD2**<br>(97-154)	**NTD3**<br>(158-222)	**CD**<br>(281-627)	**CTD1**<br>(656-755)	**CTD2**<br>(759-806)

**Dali** top hit*<br>(PDB ID, nres)<br>aligned region (domain) in the matching structure<br>Z-score, RMSD Å/aligned Cα	*E. coli* phage CBA120 **TSP1**<br>(4oj6, 758 aa)<br>70-168 (D2)<br>Z=8.6,<br>Rmsd=3.6/79	*E. coli* phage CBA120 **TSP4**<br>(5w6h, 697 aa)<br>6-74 (D1)<br>Z=4.9,<br>Rmsd=2.4/52	*E. coli* phage CBA120 **TSP4**<br>(7rfv; 250 aa)<br>178-247 (XD2)<br>Z=10.1,<br>Rmsd=1.3/64	*A. vinelandii* mannuronan C5 epimerase 6<br>(5lw3; 381)<br>16-370<br>Z=29.6,<br>Rmsd=2.9/313<br><br>*E. coli* phage phi29 TSP gp12<br>(3suc, 767)<br>32-431<br>Z=28.6,<br>Rmsd=2.8/308	Acinetobacter secreted protease CpaA<br>(6o38, 514)<br>46-132<br>Z=5.7,<br>Rmsd=3.4/86<br><br>*E. coli* phage T4 gp9<br>(1qex; 288 aa)<br>74-167 (D2)<br>Z=4.0,<br>Rmsd=3.9/73	Tyrosine phosphatase SHP2<br>(5ehr, 485)<br>107-148<br>Z=3.0,<br>Rmsd=2.3/39
**HHpred** top hit*<br>(PDB ID, nres)<br>aligned region (domain) in the matching structure<br>Probability%, E-value	*E. coli* phage G7C TFP gp63.1<br>(4qnl, 859)<br>93-166 (D2)<br>95.33%, 0.17	------	*E. coli* phage CBA120 **TSP4**<br>(7rfv; 250 aa)<br>191-248 (XD2)<br>97.15%, 2e-03<br><br>*E. coli* phage T4 **gp10**<br>(5hx2; 602 aa)<br>303-401 (XD3)<br>84.26%, 6.8	Talaromyces leycettanus endo-polygalacturona-se<br>(6kvh, 344 aa)<br>15-328<br>99.66%, 3.1e-13<br><br>*E. coli* phage HK620 TSP<br>(4xot, 597)<br>60-412<br>99.55%, 9.23-12	------	------

**Gp533 (767 aa)**

	Head region			Body region			
	**NTD1**<br>(14-91)	**NTD2**<br>(95-149)	**NTD3**<br>(155-224)	**CD**<br>(284-610)	**CTD1**<br>(622-682)	**CTD2**<br>(690-713)	**CTD3**<br>**(**725-763)
**Dali** top hit*<br>(PDB ID, nres)<br>aligned region (domain) in the matching structure<br>Z-score, RMSD Å/aligned Cα	*E. coli* phage<br>CBA120 **TSP3**<br>(5w6f, 616 aa)<br>94-167 (D2)<br>Z=9.1,<br>Rmsd=3.3/68	*E. coli* phage<br>CBA120 **TSP4**<br>(5w6h, 697 aa)<br>340-413 (D1)<br>Z=4.9,<br>Rmsd=2.9/56	*E. coli* phage<br>CBA120 **TSP4**<br>(7rfv; 241 aa)<br>178-241 (XD2)<br>Z=9.9,<br>Rmsd=1.1/64	*A. vinelandii*<br>mannuronan<br>C5 epimerase 6<br>(5lw3; 381 aa)<br>18-376<br>Z=31.5,<br>Rmsd=2.4/294<br><br>*E. coli* phage<br>CBA120 **TSP2**<br>(5w6s, 676 aa)<br>104-438 (D3)<br>Z=31.1,<br>Rmsd=2.3/294	Salmonella<br>phage P22 TSP<br>(2xc1, 661 aa)<br>544-617<br>Z=5.2,<br>Rmsd=2.4/57	----	*E. coli* phage<br>phi92<br>colanidase TSP<br>gp150<br>(6e0w; 626)<br>470-506<br>Z=2.8,<br>Rmsd=2.4/37
**HHpred** top hit*<br>(PDB ID, nres)<br>aligned region (domain) in the matching structure<br>Probability%, E-value	*E. coli* phage<br>G7C TFP<br>gp63.1<br>(4qnl, 859)<br>102-166 (D2)<br>98.76%, 2.2e-8	*E. coli* phage<br>CBA120 **TSP1**<br>(4oj5, 776 aa)<br>5-73 (D1)<br>23.22%, 54	*E. coli* phage<br>CBA120 **TSP4**<br>(7rfv; 250 aa)<br>178-248 (XD2)<br>96.97%, 3.5e-3<br><br>*E. coli* phage T4<br>**gp10**<br>(5hx2; 602 aa)<br>303-385 (XD3)<br>84.94%, 4.0	Klebsiella<br>phage KP32<br>depolymerase<br>gp38<br>(6tku, 584)<br>36-304<br>99.76%, 6.8e-16,	*E. coli* phage<br>CBA120 **TSP2**<br>(6w4q; 972 aa)<br>641-707 (D3-D4)<br>98.34%, 5.5e-7	*E. coli* phage<br>CBA120 **TSP2**<br>(6w4q; 972 aa)<br>703-747 (D4)<br>98.2%, 1e-6	*E. coli* phage<br>CBA120 **TSP2**<br>(6w4q; 972 aa)<br>745-790 (D4)<br>98.32%, 4.5e-7

**Gp531 (895 aa)**							
	Head region				Body region		
	**NTD1** (29-91)	**NTD2** (101-153)	**NTD3** (165-229)	**NTD4** (238-300)	**CD** (401-748)	**CTD1** (751-845)	**CTD2** (848-895)
**Dali** top hit* (PDB ID, nres) aligned region (domain) in the matching structure Z-score, RMSD Å/aligned Cα	*E. coli* phage CBA120 **TSP1** (4ojp, 756 aa) 95-155 (D2) Z=6.9, Rmsd=3.3/57	*E. coli* phage CBA120 **TSP4** (5w6h, 697 aa) 5-66 (D1) Z=4.9, Rmsd=2.0/51	*E. coli* phage CBA120 **TSP4** (7rfv; 241 aa) 178-241 (XD2) Z=10.0, Rmsd=1.3/64	*E. coli* phage CBA120 **TSP4** (7rfv; 241 aa) 178-238 (XD2) Z=9.4, Rmsd=1.3/61	*A. vinelandii* mannuronan C5 epimerase 4 (2pyg, 376) 15-352 Z=29.3, Rmsd=2.1/275<br><br>*E. coli* phage phi29 TSP gp12 (3gq8, 604) 31-389 Z=27.8, Rmsd=2.4/281	Acinetobacter baumannii protease CpaA (6o38, 573) 1-94 Z=10.3, Rmsd=2.3/87<br><br>*E. coli* phage T4 gp9 (1zku; 288 aa) 67-163 (D2) Z=6.1, Rmsd=3.1/80	*E. coli* phage CBA120 **TSP3** (6nw9, 616 aa) 570-616 (D4) Z=8.7, Rmsd=1.3/47

**HHpred** top hit*<br>(PDB ID, nres)<br>aligned region (domain) in the matching structure<br>Probability%, E-value	*E. coli* phage G7C TFP gp63.1<br>(4qnl, 859)<br>90-166 (D2)<br>96.2%, 2.6e-2	-----	*E. coli* phage CBA120 **TSP4**<br>(7rfv; 250 aa)<br>191-248 (XD2)<br>97%, 3.2e-3<br><br>*E. coli* phage T4 **gp10**<br>(5hx2; 602 aa)<br>303-392 (XD3)<br>79.13%, 12	*E. coli* phage CBA120 **TSP4**<br>(7rfv; 250 aa)<br>190-248 (XD2)<br>97.4%, 1.2e-3<br><br>*E. coli* phage T4 **gp10**<br>(5hx2; 602 aa)<br>303-385 (XD3)<br>68%, 26	Arthrobacter chlorophenolicus A6 hydrolase<br>(5zl5, 445)<br>19-378<br>99.78%, 3.8e-15<br><br>*E. coli* phage CBA120 **TSP3**<br>(6nw9, 633 aa)<br>215-523 (D3)<br>99.7%, 5.3e-16	*E. coli* metalloprotease StcE<br>(4dny, 126)<br>67-123<br>73.99%, 5.8	*E. coli* phage CBA120 **TSP3**<br>(6nw9, 633 aa)<br>584-627 (D4)<br>98.94%, 1.9e-9

Gp528 (1113 aa)

	Head region				
	**NTD1**<br>(1-105)	**NTD2**<br>(109-174)	**NTD3**<br>(180-244)	**NTD4**<br>(247-306)	**NTD5**<br>(317-384)
**Dali** top hit*<br>(PDB ID, nres)<br>aligned region (domain) in the matching structure<br>Z-score, RMSD Å/aligned Cα	*E. coli* phage T4 gp34<br>(5nxh, 546 aa)<br>360-496 (P5)<br>Z=7.5, Rmsd=2.3/100	*E. coli* phage CBA120 **TSP4**<br>(7rfv; 241 aa)<br>178-241 (XD2)<br>Z=10.5, Rmsd=1.3/64	*E. coli* phage CBA120 **TSP4**<br>(7rfv; 241 aa)<br>178-241 (XD2)<br>Z=10.7, Rmsd=1.2/64	*E. coli* phage CBA120 **TSP4**<br>(7rfo; 330 aa)<br>188-247 (XD2)<br>Z=9.0, Rmsd=1.4/60	*E. coli* phage CBA120 **TSP4**<br>(7rfo; 330 aa)<br>260-330 (XD3)<br>Z=8.4, Rmsd=1.8/64

**HHpred** top hit*<br>(PDB ID, nres)<br>aligned region (domain) in the matching structure<br>Probability%, E-value	--------	*E. coli* phage CBA120 **TSP4**<br>(7rfv; 250 aa)<br>199-249 (XD2)<br>97.79%, 1.2e-4<br><br>*E. coli* phage T4 **gp10**<br>(5hx2; 602 aa)<br>157-236 (XD2)<br>84.55%, 4.9	*E. coli* phage CBA120 **TSP4**<br>(7rfv; 250 aa)<br>199-248 (XD2)<br>97.54%, 4.5e-4<br><br>*E. coli* phage T4 **gp10**<br>(5hx2; 602 aa)<br>303-385 (XD3)<br>82.11%, 5.5	*E. coli* phage CBA120 **TSP4**<br>(7rfv; 250 aa)<br>191-248 (XD2)<br>97.26%, 1.3e-3<br><br>*E. coli* phage T4 **gp10**<br>(5hx2; 602 aa)<br>303-382 (XD3)<br>75.19%, 12	*E. coli* phage CBA120 **TSP4**<br>(7rfv; 250 aa)<br>191-248 (XD2)<br>97.32%, 1.1e-3<br><br>*E. coli* phage T4 **gp10**<br>(5hx2; 602 aa)<br>303-385 (XD3)<br>70.89%, 19

	**Body region**			
	**BD1**<br>(426-481)	**BD2**<br>(485-552)	**CD**<br>(650-970)	**CTD**<br>(1006-1113)
**Dali** top hit*<br>(PDB ID, nres)<br>aligned region (domain) in the matching structure<br>Z-score, RMSD Å/aligned Cα	*E. coli* phage CBA120 **TSP4**<br>(5w6h, 697 aa)<br>5-85 (D1)<br>Z=5.2, Rmsd=2.4/57	*E. coli* phage CBA120 **TSP4**<br>(7rfv; 241 aa)<br>178-241 (XD2)<br>Z=10.1, Rmsd=1.3/64	*E. coli* phage phi29 TSP gp12<br>(3gq8, 604 aa)<br>32-446<br>Z=22.2, Rmsd=3.2/300	*Clostridium histolyticum* class I collagenase<br>(1nqd, 114 aa)<br>1-111<br>Z=6.4, Rmsd=2.9/91<br><br>E. coli phage phiX174 capsid protein<br>(2bpA, 426 aa)<br>13-157<br>Z=5.2, Rmsd=3.4/103

**HHpred** top hit*<br>(PDB ID, nres)<br>aligned region (domain) in the matching structure<br>Probability%, E-value	---------	*E. coli* phage CBA120 **TSP4**<br>(7rfv; 250 aa)<br>193-248 (XD2)<br>97.11%, 2e-3<br><br>*E. coli* phage T4 **gp10**<br>(5hx2; 602 aa)<br>303-392 (XD3)<br>64.55%, 28	*E. coli* phage CBA120 **TSP4**<br>(5w6h; 697 aa)<br>198-520 (D3)<br>91.6%, 16	---------

Gp526 (580 aa)

	**Head region**		**Body region**		
	**NTD**<br>(9-91)	**BD1**<br>(113-189)	**BD2**<br>(190-263)	**BD3**<br>(274-432)	**CTD (BD4)**<br>(449-580)
**Dali** top hit*<br>(PDB ID, nres)<br>aligned region (domain) in the matching structure<br>Z-score, RMSD Å/aligned Cα	*E. coli* phage CBA120 **TSP1**<br>(4oj6, 753 aa)<br>1-83 (D1)<br>Z=12, Rmsd=1.9/80	P. aeruginosa R1 pyocin fiber PALES_06171<br>(6cl5, 374 aa)<br>120-201<br>Z=7.4, Rmsd=2.4/71	Bacillus subtilis K(+)/H(+) antiporter subunit KhtT<br>(7agv, 163 aa)<br>1-55<br>Z=4.7, Rmsd=2.6/54<br><br>*E. coli* phage T4 gp34<br>(5nxh, 546 aa)<br>402-496<br>Z=3.1, Rmsd=2.9/54	*Pantoea stewartii* glycosidase WceF<br>(6tgf, 673 aa)<br>456-621<br>Z=10.6, Rmsd=3.6/129	*Acinetobacter baumanii* phage phiAB6 TSP<br>(5jse, 548)<br>438-547<br>Z=6.7, Rmsd=2.7/81

**HHpred** top hit*<br>(PDB ID, nres)<br>aligned region (domain) in the matching structure<br>Probability%, E-value	*E. coli* phage G7C TFP gp63.1<br>(4qnl, 859)<br>1-75 (D1)<br>80.49%, 4.9	---------	--------	--------	*E. coli* phage SU10 putative tail fiber protein gp12<br>(7z4a, 786 aa)<br>652-784<br>99.87%, 2.4e-21

Gp534 (688 aa)

	N-terminus	Central region			C-terminus
	NTD<br>(1-74)	115-188	252-314	439-491	CTD<br>(523-689)
**Dali** top hit*<br>(PDB ID, nres)<br>aligned region (domain) in the matching structure<br>Z-score, RMSD Å/aligned Cα	*E. coli* phage CBA120 **TSP3**<br>(5w6f, 616aa)<br>73-150 (D2)<br>Z=6.8, Rmsd=2.8/68	*E. coli* phage Mu TFP S<br>(5yvq, 358 aa)<br>55-137<br>Z=3.7, Rmsd=1.9/66	*P. aeruginosa* R2 pyocin membrane-piercing spike<br>(4s37, 172 aa)<br>90-142<br>Z=6.0, Rmsd=2.0/52	*E. coli* phage Mu TFP S<br>(5yvq, 358 aa)<br>247-320<br>Z=2.5, Rmsd=2.6/50	Ectodysplasin A EDA-A1<br>(1rj7, 143 aa)<br>1-139<br>Z=15.1, Rmsd=2.2/133
**HHpred** top hit*<br>(PDB ID, nres)<br>aligned region (domain) in the matching structure<br>Probability%, E-value	*E. coli* phage G7C TFP gp63.1<br>(4qnl, 859)<br>102-153 (D2)<br>54.54%, 27	----------	*E. coli* phage T2 short tail fiber gp12<br>(5lye, 322)<br>255-318<br>63.83%, 5.7	----------	----------

Gp098 (595 aa)

	N-terminus	Central region		C-terminus
		aa 136-352	aa 372-483	CTD<br>(aa 493-595)

**Dali** top hit*<br>(PDB ID, nres)<br>aligned region (domain) in the matching structure<br>Z-score, RMSD Å/aligned Cα	----------	*E. coli* phage T4<br>LTF protein gp37<br>(2xgf, 216 aa)<br>1-216<br>Z=4.4, Rmsd=3.6/107	*E. coli* phage T4<br>LTF protein gp34<br>(4uxe, 396 aa)<br>39-165<br>Z=7.3, Rmsd=2.4/103	*P. aeruginosa* R2 pyocin tail fiber<br>PAO620<br>(6ct8, 273 aa)<br>151-249<br>Z=12.1, Rmsd=2.4/96
**HHpred** top hit*<br>(PDB ID, nres)<br>aligned region (domain) in the matching structure<br>Probability%, E-value	----------	----------	*E. coli* phage P2<br>gpV<br>(3qr8, 211 aa)<br>100-184<br>91.74%, 3.7	*P. aeruginosa* R2 pyocin tail fiber<br>PAO620<br>(6cl6, 372 aa)<br>285-372<br>94.32%, 0.89

*/** - If the top hit is of cellular origin, the best structural match in phages is also given.

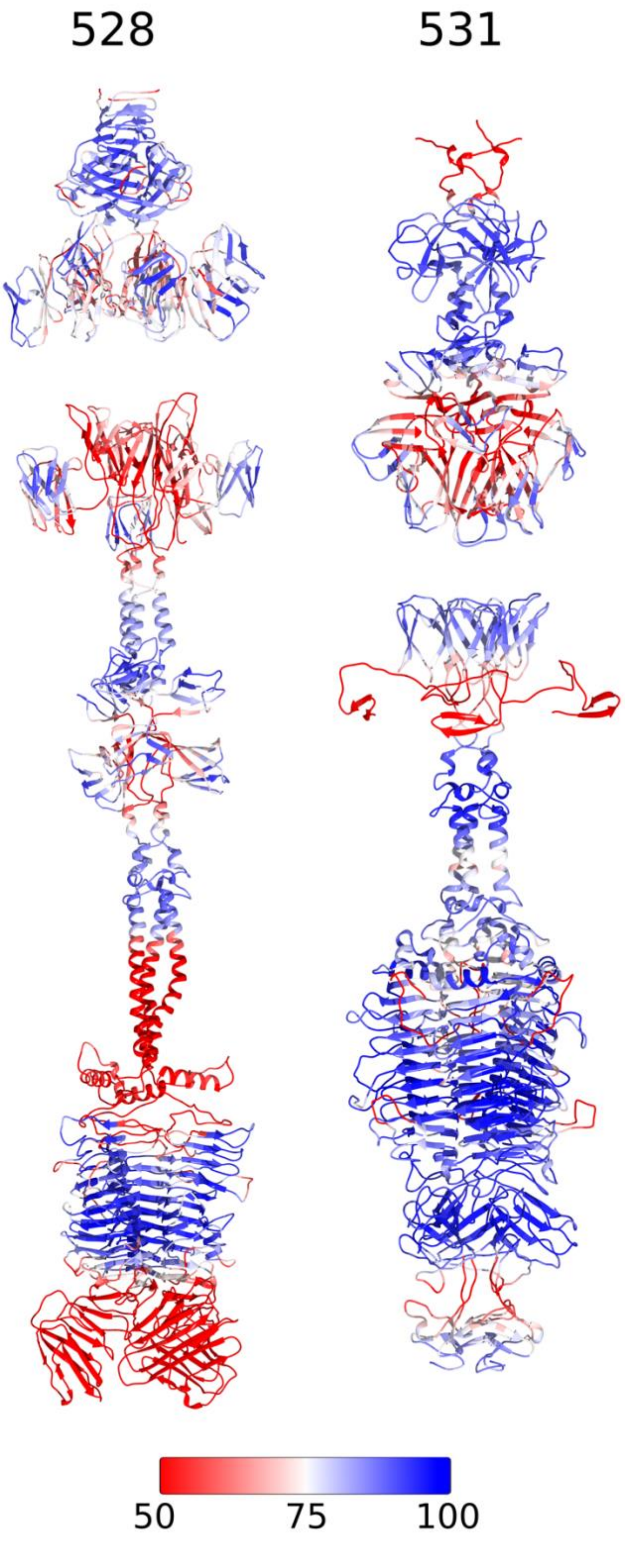

Figure S3. Predicted trimeric structures of RaK2 gp528 and gp531 colored by the pLDDT value. The color code is at the bottom center.

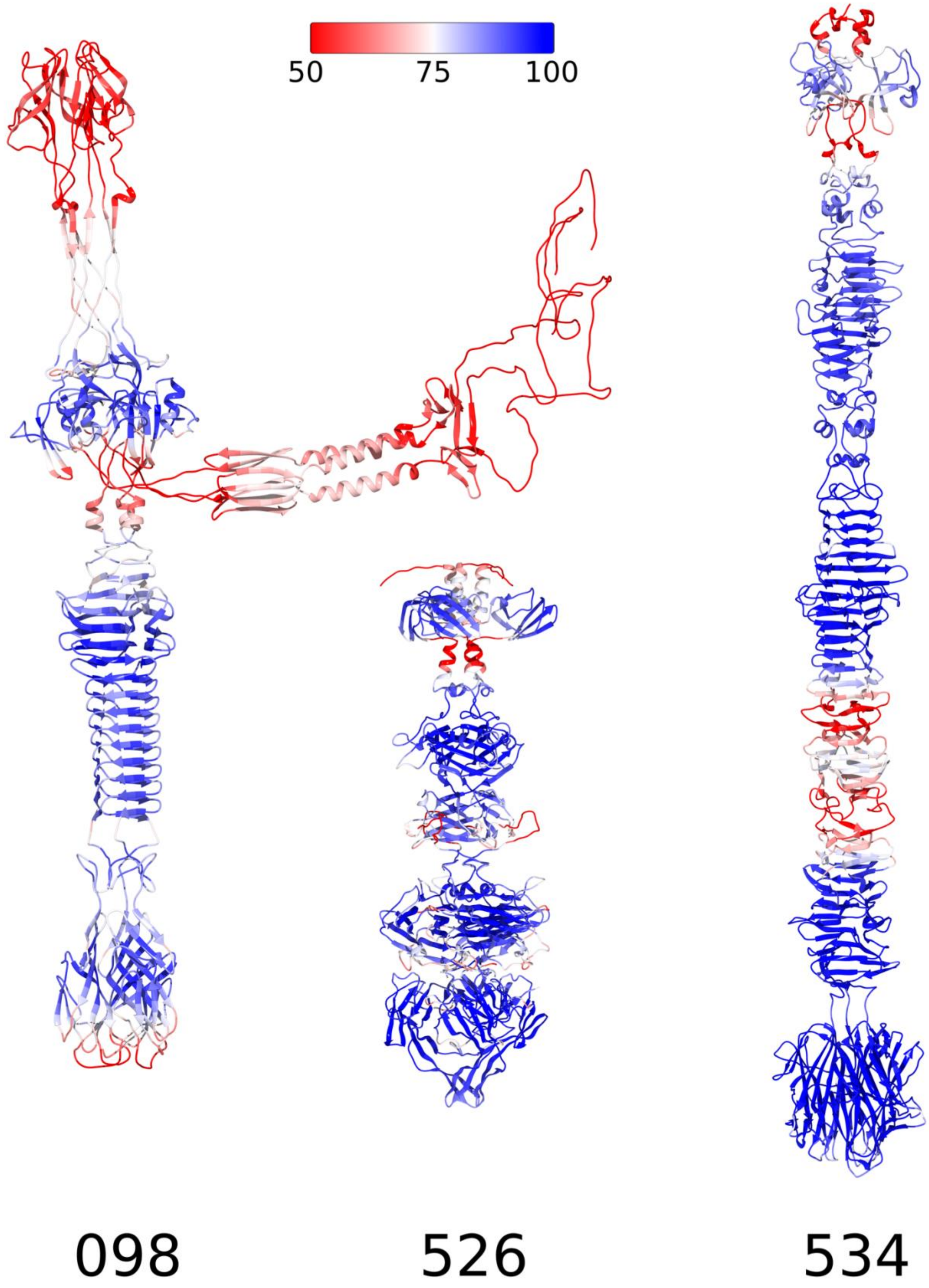

Figure S4. Predicted trimeric structures of RaK2 gp098, gp526, and gp534, colored by the pLDDT value. The color code is at the top center.

Table S3. Plasmids and primers used in this study.

Recombinant protein	Vector	RE site	F/R	Sequence (5' → 3')	Residue range
098C	pET28b	NheI	F	ATGGCTAGCGATACTATGACTGGCAAC	368-595 aa
		BamHI	R	ATTCGGATCCTGACTGTGTGTCAGAT	
526	pET28b	NheI	F	TACAGCTAGCTTAAACGAGGACAATATGTC	1-580 aa
		BamHI	R	TAACAGGATCCATTAATATTCCTAGATGTG	
527C	pET28b	NheI	F	ATGCTAGCTCTGGTACAACAAATACAA	539-715aa
		BamHI	R	TAACAGGATCCTCGTTTTAATTAGTTGG	
528N	pET16b	NdeI	F	TACACATATGAAAAGGAATTATGACATGGC	1-372 aa
		BamHI	R	TACTAGGATCCTGTAGTGGTGAGCTTAATG	
529	pET28b	NheI	F	TGGCTAGCATGGGAAATTTTATAC	1-584 aa
		BamHI	R	TCGGATCCTATTATGCACCTCTAATA	
530C	pET28b	NheI	F	GAAGCTAGCGTCAGTTACACTACATCA	538-779 aa
		BamHI	R	AAGTGGATCCTTTAGGTTGTATAAAATT	
531	pET16b	SalI	F	CTTGTCGACGAGGTTTAATATGTCATTGA	1-895 aa
		BamHI	R	TAAGGATCCTTTTTTATACTGAAGTTCCTG	
532	pET16b	SalI	F	TCAGTCGACGTCTTTAAGTAATTTAAGCTC	1-806 aa
		BamHI	R	TAATGGATCCATTACTAGGTGAAAG	
533	pET16b	SalI	F	TCAGTCGACGTCATTAATTCAACTTTCACC	1-767 aa
		BamHI	R	GCCGGATCCGATAATGACATAATCGAT	
534C	pET28b	NheI	F	CCAACGCTAGCATGCAAATAGCTGG	442-688 aa
		BamHI	R	TACAGGATCCATTATATAGTTAAGAAACTTAC	
